# Target Genes of the MADS Transcription Factor SEPALLATA3: Integration of Developmental and Hormonal Pathways in the *Arabidopsis* Flower

**DOI:** 10.1371/journal.pbio.1000090

**Published:** 2009-04-21

**Authors:** Kerstin Kaufmann, Jose M Muiño, Ruy Jauregui, Chiara A Airoldi, Cezary Smaczniak, Pawel Krajewski, Gerco C Angenent

**Affiliations:** 1 Business Unit Bioscience, Plant Research International, Wageningen, The Netherlands; 2 Institute of Plant Genetics, Polish Academy of Sciences, Poznań, Poland; 3 BIOBASE GmbH, Wolfenbüttel, Germany; 4 Centre for Plant Sciences, University of Leeds, Leeds, United Kingdom; 5 Centre for BioSystems Genomics (CBSG), Wageningen, The Netherlands; Max Planck Institute for Developmental Biology, Germany

## Abstract

The molecular mechanisms by which floral homeotic genes act as major developmental switches to specify the identity of floral organs are still largely unknown. Floral homeotic genes encode transcription factors of the MADS-box family, which are supposed to assemble in a combinatorial fashion into organ-specific multimeric protein complexes. Major mediators of protein interactions are MADS-domain proteins of the SEPALLATA subfamily, which play a crucial role in the development of all types of floral organs. In order to characterize the roles of the SEPALLATA3 transcription factor complexes at the molecular level, we analyzed genome-wide the direct targets of SEPALLATA3. We used chromatin immunoprecipitation followed by ultrahigh-throughput sequencing or hybridization to whole-genome tiling arrays to obtain genome-wide DNA-binding patterns of SEPALLATA3. The results demonstrate that SEPALLATA3 binds to thousands of sites in the genome. Most potential target sites that were strongly bound in wild-type inflorescences are also bound in the floral homeotic *agamous* mutant, which displays only the perianth organs, sepals, and petals. Characterization of the target genes shows that SEPALLATA3 integrates and modulates different growth-related and hormonal pathways in a combinatorial fashion with other MADS-box proteins and possibly with non-MADS transcription factors. In particular, the results suggest multiple links between SEPALLATA3 and auxin signaling pathways. Our gene expression analyses link the genomic binding site data with the phenotype of plants expressing a dominant repressor version of SEPALLATA3, suggesting that it modulates auxin response to facilitate floral organ outgrowth and morphogenesis. Furthermore, the binding of the SEPALLATA3 protein to *cis*-regulatory elements of other MADS-box genes and expression analyses reveal that this protein is a key component in the regulatory transcriptional network underlying the formation of floral organs.

## Author Summary

Most regulatory genes encode transcription factors, which modulate gene expression by binding to regulatory sequences of their target genes. In plants in particular, which genes are directly controlled by these transcription factors, and the molecular mechanisms of target gene recognition in vivo, are still largely unexplored. One of the best-understood developmental processes in plants is flower development. In different combinations, transcription factors of the MADS-box family control the identities of the different types of floral organs: sepals, petals, stamens, and carpels. Here, we present the first genome-wide analysis of binding sites of a MADS-box transcription factor in plants. We show that the MADS-domain protein SEPALLATA3 (SEP3) binds to the regulatory regions of thousands of potential target genes, many of which are also transcription factors. We provide insight into mechanisms of DNA recognition by SEP3, and suggest roles for other transcription factor families in SEP3 target gene regulation. In addition to effects on genes involved in floral organ identity, our data suggest that SEP3 binds to, and modulates, the transcription of target genes involved in hormonal signaling pathways.

## Abstract 3

The key floral regulator SEPALLATA3 binds to the promoters of a large number of potential direct target genes to integrate different growth-related and hormonal pathways in flower development.

## Introduction

In contrast to animals, most developmental processes of plants occur postembryonically and integrate a variety of internal and environmental cues. Modularity of plant development is based on the ability of plants to maintain pools of undifferentiated stem cells throughout the life cycle of the plant. Stem cells near the tip of the growing shoot are located in the shoot apical meristem, where different types of plant organs, such as vegetative leaves or floral organs, can be initiated from the flanks of the meristem. Leaves and floral organs are “variations of a theme”: they arise via modifications of a common basic genetic program [1]. Which type of organ is produced by the meristem depends on the developmental phase of the plant. Initially, leaves are produced during the early vegetative phase of the plant, followed by the transition to reproductive phase, which triggers the transformation of vegetative shoot meristems to inflorescence and floral meristems, giving rise to flowers and floral organs, respectively.

Thus, change in the identity of plant organs is initiated by reprogramming within the meristems [2]. Plant developmental biologists have identified a number of key regulatory genes that trigger changes in meristem and organ identity, many of them encoding transcription factors, chromatin remodeling factors, or other signaling molecules like microRNAs (miRNAs). One family of transcription factors that is important in this process is the MADS-box gene family [3]. MADS-box genes play crucial roles in the switches from vegetative to inflorescence and finally to floral meristems. These latter meristems give rise to flowers and floral organs, respectively [4].

Developmental transitions and organ differentiation require global changes in gene expression. The genome of the model flowering plant Arabidopsis thaliana is roughly 20-fold smaller than the human genome; still, it encodes about 27,000 protein-coding genes, which is more than found for humans (http://www.arabidopsis.org; [5]). One of the most challenging current questions is how developmental control genes trigger global changes in gene expression during the multiple phase transitions and in organ identity determination, starting from a small pool of undifferentiated cells.

In the present study, we focus on the MADS-box transcription factor SEPALLATA3 (SEP3). SEP3 is a member of the SEP subfamily of MADS-box genes, whose members have nearly redundant functions in the specification of floral meristem identity and in the identity of all types of floral organs: sepals, petals, stamens, and carpels. Triple mutants impaired in *SEP1–3* function have flowers with floral organs converted into sepals and display a loss of determinacy in the center of the flower [6]. This phenotype masks the involvement of the *SEP* genes in processes occurring later in development, e.g., the formation of the ovules as has been shown by Favaro et al. (2003) [7]. The SEP3 protein appears to be the central player, since it is part of at least a dozen different MADS domain dimer complexes [8] and it is expressed throughout flower development, from the floral meristem to fully developed floral organs [9]. This suggests that SEP3 is a multifunctional protein controlling a plethora of developmental processes. According to the current model of flower development, the SEP3 protein is proposed to mediate the higher-order protein complex formation between MADS-domain proteins with more specific floral organ identity functions [10]. Furthermore, it may provide the transcriptional activation potential to the floral homeotic protein complexes [10]. More-recent evidence suggests that the SEP3 protein may also recruit transcriptional corepressors, demonstrating that it can modulate the function of the plant protein complexes in a broader sense, depending on the availability of cofactors [11]. However, evidence for higher-order complex formation between MADS-domain proteins comes mostly from protein interaction studies in heterologous systems and genetic data, and there is no indication for the relevance of these interactions in target-gene recognition *in planta* so far. Another question is how different MADS-domain protein complexes achieve functional specificity, since the in vitro DNA-binding characteristics of MADS-domain proteins appeared rather similar, and the short DNA sequence motifs supposedly bound by MADS-domain proteins are very abundant in the *Arabidopsis* genome [12].

In order to characterize the mode of action and general downstream pathways of floral homeotic genes, we generated genome-wide DNA-binding profiles of SEP3 in its native context. Chromatin immunoprecipitation (ChIP) followed by ultrahigh-throughput Solexa (Illumina) sequencing (ChIP-SEQ) has been shown recently to be a powerful tool to obtain genome-wide DNA-binding patterns of transcription factors [13,14]. The large numbers of short individual sequence reads produced by novel instruments facilitate the digital quantification of DNA sequences that are present in a sample. An alternative method comprises the combination of ChIP and whole-genome microarrays (ChIP-CHIP) to map the genomic DNA regions enriched in the immunoprecipitated sample [15,16]. These genomic tiling arrays are available for *Arabidopsis* and have been used to map binding sites for plant transcription factors [17].

We compared the targets of SEP3 in wild-type and the floral homeotic *agamous* (*ag*) mutant background. In the *ag* mutant, stamens are replaced by petals, and instead of the carpels in the fourth whorl, a new mutant flower is formed [18]. Accordingly, the analysis of this mutant should reveal SEP3 target genes specifying petal development, whereas targets that are specific to stamens and carpels should be absent. We further studied the function of SEP3 in the regulation of downstream pathways by analyzing the effects of a dominant repressor version of SEP3 in plants.

The genome-wide identification of direct target genes of SEP3 provides a framework for a hierarchical transcriptional network underlying the formation of floral organs. SEP3 binds to thousands of genomic regions containing the consensus binding sites for MADS-domain proteins, but it also acts as part of regulatory modules with other transcription factors. These modules link floral homeotic gene functions with organ growth. Our analysis identified multiple links between SEP3 and hormonal pathways, and in particular auxin signaling. Auxin signaling is crucial for the outgrowth and development of lateral organs, and its role in flower development has been suggested previously based on mutant phenotypes [19–21]. Our ChIP-SEQ data and the phenotypes of plants that repress direct SEP3 targets in a dominant-negative fashion suggest cooperation of SEP3 and genes in the auxin pathway in the regulation of floral organ growth and differentiation.

## Results

### Genome-Wide Mapping of Regions Bound by SEPALLATA3

To identify genomic regions bound in vivo by SEPALLATA3 (SEP3), we used ChIP followed by deep sequencing by ChIP-SEQ [14,15]. In a parallel experiment, the ChIP was followed by whole-genome tiling array hybridizations to identify enriched regions (ChIP-CHIP). For the ChIP experiments, we used inflorescences including inflorescence meristems and floral buds of stage 1–12 from *Arabidopsis* wild-type and *agamous* (*ag*-1) mutant plants. Protein–DNA complexes were immunoprecipitated using a peptide antibody specific to SEP3. As a negative control, we performed ChIP-SEQ using the same antibody on *sep3*-1 mutant plants. Western blot analysis revealed that the SEP3 antibody reacts exclusively with the SEP3 protein (Figure S1).

From the ChIP-SEQ experiments, we obtained between 3 to 7.5 million approximately 35-bp sequence reads after one to three independent rounds of sequencing for each sample, of which 30%–40% were uniquely mapped on the *Arabidopsis* nuclear genome (Table S1). The uniquely mapped reads were extended to 300 bp in order to recover the average original DNA fragments that were subjected to sequencing in a similar fashion as described by Robertson et al. (2007) [14]. This allows positioning of the maximum of enrichment present in the samples at high resolution. The number of mapped reads was counted for every nucleotide position (defined as “number of hits”), and for each strand independently. For some genomic positions, we observed that the number of hits was only due to reads with identical sequence. Although, it is expected that some reads will have an identical sequence, it is also expected that a true peak of enrichment should be represented by several reads with partly overlapping but different sequences. In order to avoid any artifact due to identical sequence reads, we included the requirement that the number of hits at each genomic position should be supported by reads mapped in both DNA strands. To test for enrichment at each nucleotide position in the sample compared to the control, we used a score based on the Poisson distribution, as it is commonly used for statistical modeling of tag counts [22]. For each genomic region representing a candidate peak, the maximum score value was used to test the significance of the peak (defined as “peak score”). We used the false discovery rate (FDR) to control the error rate of our testing procedure.

The number of significant peaks for the ChIP-SEQ datasets is given in Table 1. Notably, SEP3 binds to thousands of regions in the *Arabidopsis* genome. At FDR <0.001, we found 4,282 significantly enriched regions for the SEP3 in wild-type plants, and 2,828 regions in the *ag* mutant. Thus, at this level of significance and given our data, SEP3 seems to bind a reduced number of regions in the *ag* mutant compared to wild-type plants.

**Table 1:**
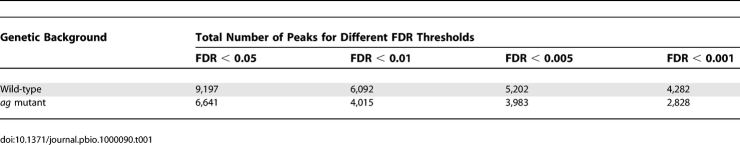
Number of Significant Peaks in the SEP3 ChIP-SEQ Datasets

We used biological replicates and comparison of ChIP-SEQ and ChIP-CHIP data in order to evaluate the reproducibility of the generated genome-wide binding profiles of SEP3. To simplify the comparison of ChIP-SEQ replicates, the average score in nonoverlapping 5,000-bp windows for each replicate was calculated. A high correlation between the two different sequencing rounds and biological replicates was found (Figure S2E and S2G). Peaks positions and ranks from independent biological replicates overlap strongly (Figure S2H). Since more sequences were produced for replicate 1, we focused in our further bioinformatic analysis on this replicate.

Comparison of the ChIP-CHIP and the ChIP-SEQ experiments reveals a good agreement between the results of the two methods, as reflected in the large overlap in peak positions and similar ranking of the peaks (Figure S2). We were also interested in comparing the positional resolution of the two platforms. The average width of the most strongly significant ChIP-SEQ peaks is around 800 bp. In contrast, the peaks with an equivalent ChIP-CHIP rank have a width of approximately 1,300 bp (Figure S2). This larger window size for the ChIP-CHIP peaks compared to ChIP-SEQ peaks results in a lower positional resolution, which is particularly problematic in regions with multiple binding sites that are close to each other.

### Genome-Wide Overview of Bound Regions

*cis*-Regulatory elements controlling gene expression are preferentially found in the promoters of target genes. However, there are also numerous examples of important regulatory sequences in introns, particularly near the 5′ end of genes [23]. A typical example is the second intron in the MADS-box gene *AG*, which is bound by multiple factors [24–26]. AG, on its turn, binds to the downstream region of the *SPOROCYTLESS* gene, demonstrating that *cis*-regulatory elements are not exclusively located in the upstream or intragenic regions of plant genes [27].

We determined the position of the putative binding sites relative to the nearest gene based on our ChIP-SEQ dataset. As evident from our ChIP-SEQ experiments, most in vivo binding sites of SEP3 are close to or within protein-coding genes and only about 6% to 8% of all peaks (FDR <0.001) are not located within 3 kb upstream to 1 kb downstream of any genomic locus (wild-type and *ag* datasets). A total of 3,475 genes are targeted by SEP3 in wild type, whereas 2,424 genes are putative targets in the *ag* mutant at FDR <0.001 (Tables S2 and S3). In agreement with its role as transcriptional regulator, DNA-binding sites of SEP3 are predominantly located in the upstream region of genes (Figure S3; Table S3). Notably, we found the highest enrichment of SEP3 binding sites in a region spanning a few hundred base pairs directly upstream of the annotated transcriptional start of genes (Figure S3). Surprisingly, binding sites are also enriched in the downstream region of genes, located just downstream of the 3′ UTR, although this enrichment is clearly less pronounced than the one found near the transcriptional start site of genes. Within genes, peaks are preferentially located in introns and UTR regions (Figure S3 and Table S3).

### Position of MADS-Domain Binding Motifs

It is known from in vitro–binding studies that MADS-domain proteins bind to specific DNA elements called CArG boxes (reviewed in [12]). Most well known is the serum response element (SRE or SRF)-type CArG box, which has the consensus CC[A/T]_6_GG. A related sequence motif is the MEF2-type CArG box, which has the general consensus C[A/T]_8_G but is usually more strictly defined as CTA[A/T]_4_TAG. Plant MADS-domain proteins often show relatively broad DNA-binding preferences, recognizing SRF^−^ and MEF2^−^, as well as intermediate motifs [28,29]. CArG boxes are frequently found in the *Arabidopsis* genome, so the presence of this motif alone is not sufficient to predict targets of MADS-box transcription factors [12]. Given the large number of MADS-box transcription factor genes present in the *Arabidopsis* genome and the capacity to form an even larger number of heterodimeric transcription factor complexes [8], and considering their divergent functions in plant development, it is important to understand how different MADS-domain proteins (and protein complexes) achieve target-gene specificity. Also, the relevance of the formation of higher-order protein complexes in DNA binding, associated with the binding to more than one CArG box, has not been demonstrated *in planta* yet.

**Figure 1:**
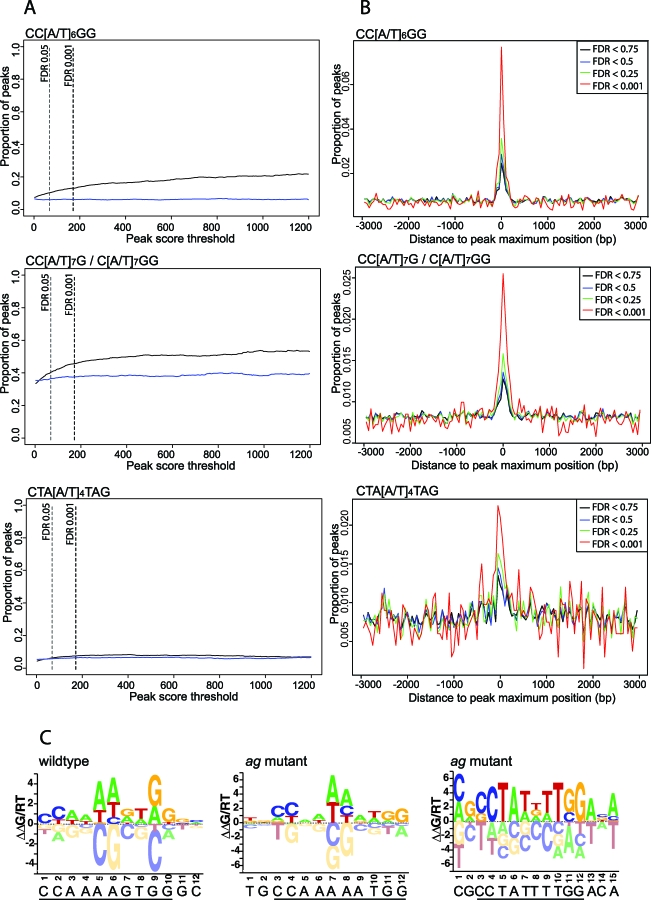
Enrichment, Position, and Sequences of CArG Boxes in ChIP-SEQ Peaks (A) Enrichment of CArG boxes in peak areas with increasing significance threshold. All different types of CArG boxes show an increase in frequency with increasing significance level. Note that the frequency of the MEF2-type CArG box increases only very weakly enriched. Black line: proportion of peaks with at least one binding site. Blue line: proportion of peaks with at least one binding site after permutating the nucleotide positions of the peak areas, keeping the same base composition (i.e., control). (B) Frequency plots of sequence position of CArG boxes relative to the peak score maximum position (“center of the peak”). Only perfect CArG boxes were considered in the analyses (no mismatches allowed). All three types of CArG boxes show enrichment in the center of the peak, with the canonical SRF motif CC[A/T]_6_GG displaying the strongest enrichment in the peak maximum score position. (C) Affinity logos of CArG box–like elements identified by MatrixREDUCE [30]. The logo represents the estimated DNA-binding affinity (ΔΔG) for each nucleotide position. One consensus motif resembling a CArG box was identified for the wild-type ChIP-SEQ data, whereas two motifs were found in the *ag* mutant data.

We considered the 1,001 bp surrounding the peak maximum score position, defined as peak area, for the characterization of transcription factor binding sites. The peak areas were searched for the presence of different types of CArG boxes. Our results show that the SRF, MEF2, and intermediate types are enriched in the genomic regions bound by SEP3 in vivo (Figure 1A). In agreement with a true enrichment of CArG boxes in genomic regions bound by SEP3, we found that the “background” frequency of CArG boxes of type CC[A/T]_6_GG in promoter regions (−1,000 bp upstream) was approximately 7%, whereas in our ChIP-SEQ data at FDR = 0.001, the frequency is approximately 12.5%; and in more strongly bound regions, the frequency increases to more than 20%. SRF and intermediate types of CArG boxes showed considerably more enrichment than MEF2-type CArG boxes, suggesting that they are more frequently bound by SEP3 or SEP3-containing protein complexes *in planta*.

The CArG boxes are usually positioned in the center of the peak (peak maximum score position) (Figure 1B). The strong preference of CArG boxes for the center of the peak demonstrates the high positional resolution of ChIP-SEQ experiments combined with our method of peak detection.

In order to further characterize the features of binding sites recognized by SEP3 and its complex partners *in planta*, we used the ChIP-SEQ peak score as a measure of affinity for binding sites within enriched genomic regions as implemented in the MatrixREDUCE software [30]. We found that the obtained consensus models tend to be relatively flexible in most nucleotide positions (Figure 1). This suggests that the in vivo binding data reflect a mix of affinities of different homo- and heterodimeric SEP3 protein complexes.

Since MADS-domain proteins bind as dimers to a CArG box, CArG boxes can be considered as composite elements, with each half-site contacted by a different monomer with possibly different binding preferences. We estimated the frequencies of all possible half-sites for the CArG boxes of the general consensus CC[A/T]_6_GG and CC[A/T]_7_G in the ChIP-SEQ data. Using a binomial test, we found that three out of eight possible half-sites for the consensus CC[A/T]_6_GG were significantly overrepresented in the SEP3 ChIP-SEQ data, and four out of 24 for the consensus CC[A/T]_7_G (Table S4). Not all possible combinations of the most frequent half-sites are represented among the most strongly overrepresented core CArG boxes (Table S4), suggesting dependencies between the half-sites. The most frequently represented sequence of type CC[A/T]_6_GG, which is CCAAAAATGG, is in fact the same or highly similar to the consensus sequences that were identified by MatrixREDUCE (Figure 1C).

Based on the combinations of half-site sequences found in the ChIP-SEQ data, we measured the dependencies between single nucleotides using a chi-square test. Here, we found that strong dependencies exist between nucleotides within the [A/T]-rich core of the CArG box (Table S5). Surprisingly, we also identified dependencies for the nucleotides surrounding the core CArG box, which is in line with experimental evidence suggesting that sites surrounding the core consensus are contacted by MADS-domain proteins and may contribute to DNA-binding specificity as well [31–33]. The dependencies between nucleotide positions in functional CArG boxes could (at least partly) explain why only 7.7% and 5.7% of all CArG boxes perfectly matching the consensus CC[A/T]_6_GG and C[A/T]_7_GG, respectively, are bound in vivo by SEP3.

According to the “floral quartet” model [34], higher-order MADS-domain protein complexes bind to two CArG box–like DNA sequences at short distance from each other. Thus, we would expect an enrichment of ChIP-SEQ peaks with regulatory modules consisting of two CArG box elements. In order to identify regulatory modules composed of more than one binding site, we used the Explain software [35]. The module with the highest fitness score (0.748 on a scale of 0 to 1) was composed of a single pair of CArG boxes separated by a DNA stretch varying between 10 to 200 bp in length. This finding supports the idea that MADS-domain proteins act in complexes composed of two dimers that bind to two adjacent binding sites, as has been predicted by the floral quartet model.

**Figure 2:**
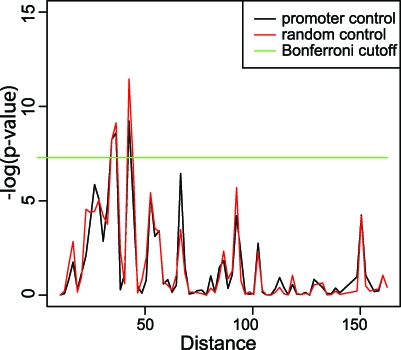
Preferred Distances between CArG Boxes within ChIP-SEQ Peaks Plotted are the −log_e_(*p*-value) resulting from a binomial test for enrichment of distances of CArG boxes in wild-type ChIP-SEQ data compared to a random set of promoters (promoter control) and randomized sequences (random control). The Bonferroni correction gives the most conservative cutoff for significantly enriched distances. The most strongly preferred distance is at 42–43 bp.

Next, we were interested whether CArG boxes within ChIP-SEQ peaks have preferred distances from each other. To test this, we compared the distribution of distances of CArG boxes within peaks to “background distributions” obtained from random sets of *Arabidopsis* promoters or randomized sequences. As shown in Figure 2, there is a preference for close distances, with the strongest preference around 42–43 bp (which corresponds to four helical turns of the DNA). Aside from these relatively short distances, the frequent occurrence of multiple peaks in the same genomic regions open the possibility that MADS-domain protein complexes can also bridge and bend larger DNA stretches as was suggested in the floral quartet model [34].

### Binding Sites for Other Transcription Factors in the ChIP-SEQ Peaks

**Figure 3:**
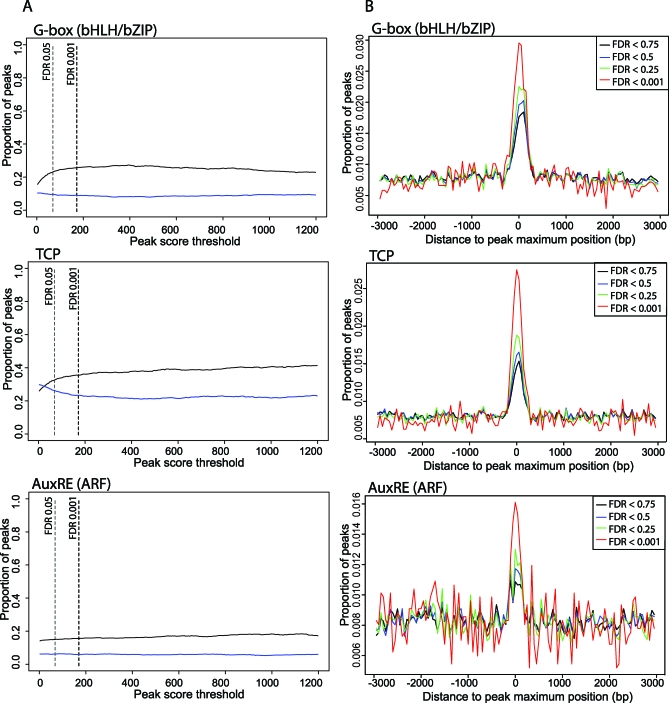
Non–MADS-DNA Binding Motifs Enriched in ChIP-SEQ Peaks (A) Increase in frequency of the motifs with increasing significance level (peak score threshold). Black line: proportion of peaks with at least one binding site; blue line: proportion of peaks with at least one binding site after permutating the nucleotide positions of the peak areas, keeping the same base composition (i.e., control). (B) Frequency plots of sequence position of TCP, bHLH/bZIP, and ARF binding sites relative to the peak score maximum position (“center of the peak”) (no mismatches allowed). All sequence elements show enrichment in the center of the peak.

In addition to our targeted screen for MADS-domain binding sites, we used MatrixREDUCE and MEME [36] in order to identify DNA sequence motifs that are abundant in the regions bound by SEP3. Using these tools, we recovered motifs corresponding to CArG boxes, but interestingly, we also detected sequence motifs potentially bound by non-MADS transcription factors. A motif that was identified using these programs has the consensus sequence CACGTG. This motif has been named “G-box” in the literature and represents a DNA-binding site for bHLH and bZIP transcription factors [37]. We found that this motif is indeed overrepresented in the genomic regions bound by SEP3 (Figure 3A). Similar to CArG boxes, the G-box motif is enriched in the center of the peak (Figure 3B). A second motif that was found using these programs has strong similarity to the DNA-binding consensus of TCP transcription factors with the general consensus motif CCNGGG [38]. We analyzed whether this TCP DNA binding consensus was overrepresented in the regions bound by SEP3. Indeed, it appeared to be enriched with increasing peak score threshold, and it is enriched in the center of the peak (Figure 3A and 3B).

Next, we tested systematically for enrichment of known DNA-binding consensus sequences of transcription factors using information from the Transfac and AGRIS databases. In total, these databases contain information for 105 (Transfac) and 72 (AGRIS) DNA-binding consensus sequences of plant transcription factors. In addition to confirming the enrichment of MADS-, TCP, and bHLH/bZIP binding sites, we found that also ARF, C2H2 (ID1) DNA recognition motifs, and a bHLH (MYC) DNA-binding site similar to the G-box were enriched with increasing peak score threshold, and located in the center of the ChIP-SEQ peaks (Figures 3 and S4).

### Overlap of SEP3 Binding Sites in Wild-Type and *agamous* Mutant Backgrounds

**Figure 4:**
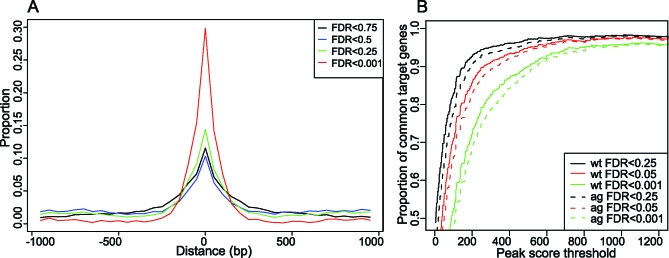
Distance between Peaks in Wild Type and *agamous* Mutant and Overlapping Target Genes (A) Proportion of distances between significant peaks in wild type and *ag* mutant for different FDR levels. Most peaks cluster within a distance of ±200 bp. (B) Proportion of common target genes for wild-type and *ag* mutant datasets. The solid line represents the proportion of common targets genes relative to the total number of targets in the *ag* mutant for different FDR levels of the wild-type dataset; the dashed line represents the proportion of common target genes relative to the total number of targets in the wild-type for different FDR levels of the *ag* mutant dataset. Since the total number of significant peaks is lower in *ag* mutant than in the wild-type dataset, the dashed line is below the solid line.

The ChIP-SEQ experiments were done with samples from wild-type plants and the *ag* mutant. We were interested in the overlap of DNA-binding sites in these two samples, which could point to target genes involved in the formation of perianth organs. There is clear preference for overlapping genomic positions of SEP3 ChIP-SEQ peak maximum positions in wild type and *ag* mutant (Figure 4A).

The overlap of potential SEP3 targets in wild type and *ag* mutant is also evident from genes that are targeted by SEP3 (Figure 4B). Whereas the number of peaks in *ag* is only approximately 65% of the number of peaks in wild type at FDR = 0.001, the overlap in affected target genes is almost 70% at the same FDR level (Figure 4B). Thus, individual peaks are more likely affected by loss of AG than target genes. With increasing significance level, targets of SEP3 in wild type and *ag* mutant overlap progressively more (Figure 4B), as do individual peaks. Highly enriched target genes are usually common to wild type and *ag* mutant (overlap >90%).

According to these results, only a small fraction of strongly enriched direct target genes is specific to the SEP3 complexes specifying stamen and carpel development (Table S2). These genes may represent candidate genes determining the specific morphologies of stamens and carpels downstream of the floral homeotic genes.

### Expression of Candidate SEP3 Targets during Flower Development

In the ChIP-SEQ approach to identify potential SEP3 targets, different floral tissues corresponding to different developmental stages were used. In order to evaluate the relevance of DNA-binding events in the regulation of the genes corresponding to the ChIP-SEQ peaks, we used comprehensive gene expression array data that are publicly available. Mainly, the collection of AtGenExpress experiments provides information about timing of gene expression and changes in different floral homeotic mutants [39].

We found that about 45% of all genes with significantly enriched peaks in the SEP3 ChIP-SEQ experiment (FDR <0.001) were differentially expressed at very young developmental stages in at least one of the homeotic mutants (*lfy-*12, *ap1-*15, *ap2-*6, *ap3-*6, *ag-*12) (Figure S5). This fraction was higher than the overall genome-wide fraction of differentially expressed genes in these mutants (29%). Forty-five percent (903/2022) of the genes with ChIP-SEQ peaks in the *ag* mutant are differentially expressed in the *ag*-12 mutant compared to wild type (up to floral stage 12; 28% (5,927/21,039) in the total dataset). Considering SEP3 binding sites in genes that are differentially expressed during development, we found the strongest enrichment for genes that change expression in the meristem during the earliest stages of floral development (FDR <0.001, *p*-value 4.3e−40, binomial test). About 63% of the potential targets of SEP3 are differentially expressed at any stage of reproductive development starting from floral transition to flowers of stage 12 (Figure S5).

In total, 72% of the potential SEP3 targets are differentially expressed during flower development or in any of the homeotic mutants. Although the differential expression can also be due to indirect effects, the data suggest that the majority of potential direct SEP3 targets may also be regulated by SEP3. We also found an enrichment in frequency of genes that are correlated in expression with SEP3 expression with increasing peak score threshold in ChIP-SEQ (Figure S5). The fraction of genes with ChIP-SEQ peaks that are positively coexpressed with SEP3 is clearly higher than that of negatively regulated genes, supporting the idea that SEP3 acts mostly as a transcriptional activator.

### Evidence for Direct Regulatory Interactions among MADS-Box Transcription Factors

Genetic and gene expression experiments suggest that the *SEP* genes are required for the up-regulation of floral homeotic genes, and that this up-regulation is crucial for the establishment of the identities of the different floral organs. However, until now, it has not been demonstrated whether this regulation is direct.

**Figure 5:**
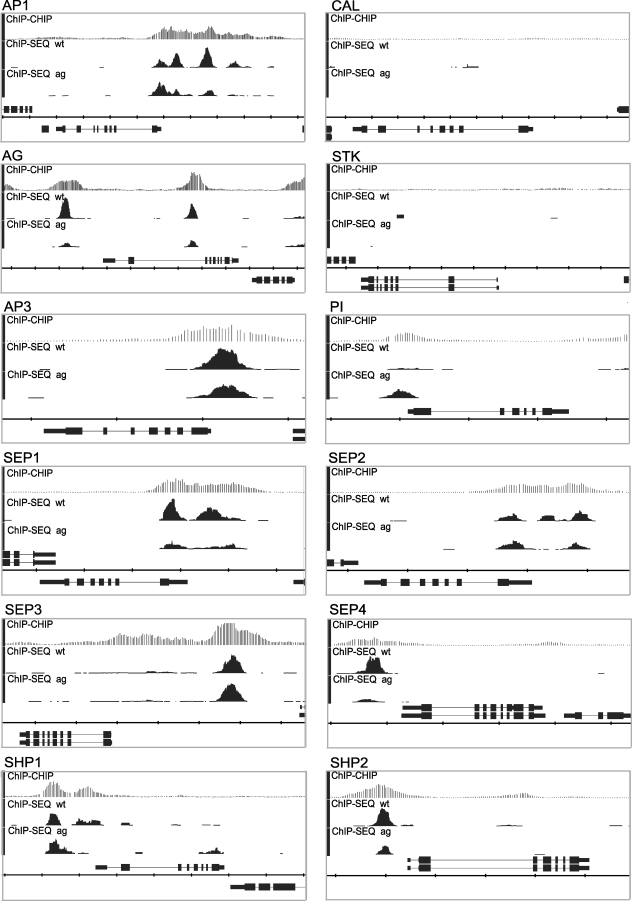
Binding Profiles of Floral Homeotic MADS-Box Gene Loci For each locus, ChIP-CHIP and ChIP-SEQ profiles are depicted for SEP3 in wild type (wt) and in the *ag* mutant. The TAIR annotation of the genomic loci is shown at the bottom of each panel. If the genomic locus is shown above the scale, it is in forward orientation, and if it is in the bottom of the scale, it is in reverse orientation. The scale division corresponds to 1,000 nt. In most cases, the enrichment is in the upstream regions of the respective genes.

We analyzed the binding profiles for the genomic loci corresponding to the floral homeotic genes and found that SEP3 binds to nearly all of these loci (Figure 5). Only *SEEDSTICK* (*STK*) and *CAULIFLOWER* (*CAL*) do not have significantly enriched regions. In most cases, the peaks are located in the promoters of the respective genes. In case of the *APETALA1* (*AP1*)*, APETALA3* (*AP3*), *SEP1*, and *SEP2* loci, there are also peaks in the 5′ UTR, whereas a SEP3 binding site is present in the second intron of *AGAMOUS* (*AG*).

Of all homeotic genes, the genomic regulatory sequences controlling the expression of *AG* and *AP3* are best characterized. The spatial expression pattern of *AP3* is driven by regulatory elements within approximately 500 bp upstream of the transcriptional start. CArG boxes in this part of the promoter are important for the positive as well as negative regulation of *AP3* [40]. Our ChIP-SEQ results demonstrate that SEP3 binds to the genomic region comprising positively and negatively acting CArG boxes in the *AP3* promoter, strongly suggesting a direct molecular link between the binding of SEP3 and the regulation of *AP3*.

Most regulatory sequences controlling the expression of *AG* are located in its second, 4-kb large intron [24,25]. Consistent with this observation, we identified a peak of enrichment of SEP3 in this intron. More specifically, the peak marks a CArG box in the 3′ activation domain located in this intron. The 3′ activation domain functions in the up-regulation of *AG* in stage 3 floral meristems and is also responsible for maintenance of *AG* expression in developing carpels [24]. This CArG box was also found to be bound by AG itself in previous experiments [26]. Interestingly, we identified a second peak of enrichment in the upstream region of *AG*. Consistent with the idea that an AG/SEP heterodimer is responsible for the positive autoregulation of *AG* [26], the heights of the peaks in the *AG* locus are reduced in the *ag* mutant compared to wild type (Figure 5).

The regulatory sequences controlling the expression of the *SEP1–4* genes are still not well characterized. Our ChIP-SEQ results, however, strongly suggest autoregulation of the redundantly acting *SEP* MADS-box genes.

All floral MADS-box genes that are targeted by SEP3 in wild type, are also targeted in the *ag* mutant, although there is some variation in the heights or presence of individual peaks (e.g., *AP1*, *SEP2*, *SHP1*; see Figure 5). This raises the possibility that different SEP3 complexes may have different affinities to individual binding sites.

**Figure 6:**
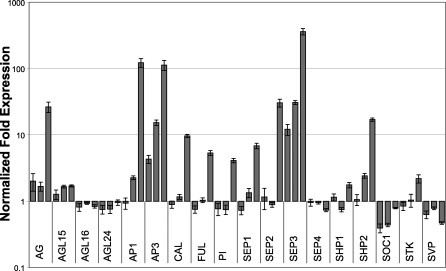
Differential Expression of SEP3 Targets of the MADS-Box Gene Family after SEP3 Induction SEP3 expression was induced in seedlings for 8 h, 1 d, or 10 d, and the change in expression of selected MADS-box genes relative to noninduced seedlings was determined by quantitative RT-PCR. The standard error represents the variation between two independent biological and technical replicates. All of the depicted genes, with the exception of *CAL* and *STK*, are bound by SEP3 (see Figure 5). In line with being indirect targets, these two genes are only up-regulated 10 d after SEP3 induction.

In order to characterize the regulatory effects of SEP3 on MADS-box genes that are potential direct targets, we analyzed the expression of these MADS-box genes upon SEP3 induction using a constitutively expressed translational fusion of SEP3 to the rat glucocorticoid receptor hormone binding domain (GR). For this, seedlings expressing the 35S:SEP3-GR construct were treated with dexamethasone (DEX) for 8 h, 1 d, or 10 d, and the expression of MADS-box genes was determined by real-time reverse transcriptase (RT)-PCR. The relative expression levels in comparison with nontreated plants is shown in Figure 6. Our results reveal that SEP3 is indeed able to activate the expression of other floral homeotic genes as suggested previously [6]. *SEP3* itself is most strongly up-regulated, demonstrating a strong autoregulatory feedback loop. Although some of the tested genes show an early response to SEP3 induction, others are regulated only after prolonged SEP3 induction, suggesting that SEP3 alone is not sufficient to regulate these genes, but needs to interact with partner proteins that are encoded by the induced MADS-box genes. In particular, *AP3*, *AG*, and *AP1* are strongly activated by SEP3. These three genes correspond to the three major classes of floral homeotic genes according to the classical ABC model: class A (*AP1*), class B (*AP3*, together with PI), and class C (*AG*). Their gene products also represent major protein interaction partners of SEP3, suggesting that later induced targets of SEP3 are targets of the corresponding SEP3-containing protein complexes. Thus, SEP3 can activate the flower developmental program by enhancing the expression of its interaction partners as one of its first steps. Induction of the ABC classes of genes is sufficient to form the flower, which explains the very early flowering and the terminal-flower phenotype that we observed upon SEP3 induction. Whereas flower-specific genes are mostly activated, MADS-box genes that are involved in the floral transition (*AGL24*, *SOC1*, and *SVP*) tend to be down-regulated by SEP3. Together with the fact that SEP3 binds to the promoters of these genes, our results suggest that SEP3 is involved in the down-regulation of these genes during early flower development, possibly as part of protein complexes together with other flower-specific MADS-domain proteins, such as AP1.

### Functional Characterization of SEP3 Target Genes

It has been a long-standing question in plant developmental biology whether floral homeotic genes act directly on the structural or metabolic genes that create the final morphology of floral organs, or whether they act via intermediate regulators, i.e., other transcription factors, which in turn regulate subsets of targets conferring the final organ shape and function. To answer this question, we investigated the enrichment of gene ontology (GO) terms [41] among genes that are closest to the peaks as a function of their ChIP-SEQ peak score.

**Figure 7:**
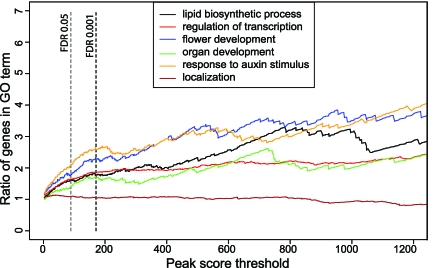
Enrichment for GO Terms in the Category “Biological Process” The five most strongly enriched GO terms of the lowest possible level (minimum 20 annotated loci) in the GO hierarchy are presented. The GO term “localization” is shown as an example for a nonenriched category.

In terms of molecular function, genes encoding transcription factors are clearly the most enriched group of genes (GO:0030528; *p*-value 3.62e−19). When dissecting gene functions according to biological processes, there is a clear enrichment for genes involved in development, in response to hormonal stimuli, and in lipid biosynthesis. Figure 7 presents the top-five most enriched specific GO terms. The SEP3 targets in the GO category “lipid biosynthetic process” include genes involved in hormone biosynthesis (terpenoid and steroid pathways), as well as in sterol and wax synthesis.

Next, we were interested in whether some transcription factor families were more frequently represented among potential direct SEP3 target genes than others. The results shown in Table 2 reveal that 15 transcription factor families were significantly overrepresented among SEP3 targets. Interestingly, we found overrepresentation of families for which we also found enrichment of their DNA-binding sites in the ChIP-SEQ data: bHLH, TCP, and ARF families are overrepresented in both the target and the binding site datasets, which points to the existence of autoregulatory feedback loops (Figure 5, Table 2). In general, we found overrepresentation of transcription factor families with known functions in the control of flowering time (SBP and C2C2-Co-like), organ growth (GRF and TCP), auxin response (AUX-IAA and ARF), brassinosteroid response (BES1), and meristem development (HB and GRAS). Sixty-six percent of the loci belonging to these families identified in wild type, were also found in the *ag* mutant at the same FDR threshold.

**Table 2:**
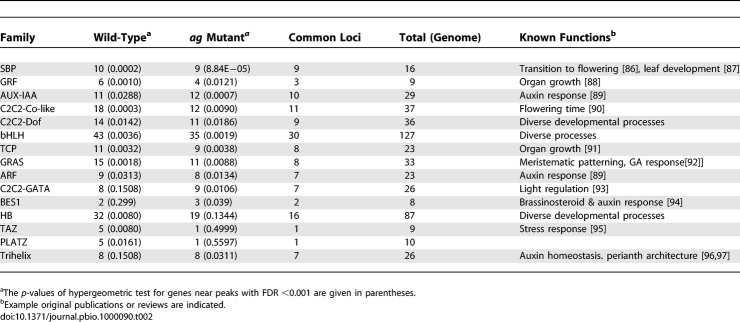
Significantly Overrepresented Transcription Factor Families

**Figure 8:**
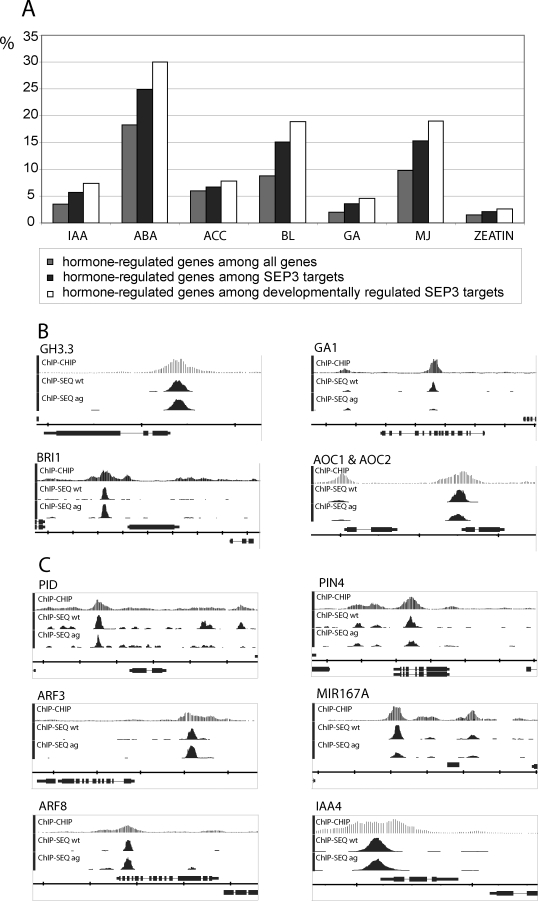
Hormonal Signaling Targeted by SEP3 (A) Characterization of hormone-regulated genes among SEP3 targets. The fraction of hormone-regulated genes among all genes represented on the ATH1 microarray is given (grey box), as well as the fraction of hormone-regulated genes among SEP3 targets (black box) and the fraction of hormone-regulated genes among SEP3 targets that are differentially expressed during reproductive development (white box). Hormone-regulated targets are overrepresented among SEP3 targets, with the strongest overrepresentation for IAA-, BL-, and GA-regulated genes (enrichment >1.6-fold among all SEP3 targets and >2.1-fold among developmentally regulated targets). Data on hormone-responsive genes (low stringency) were taken from [42]. Developmentally regulated SEP3 target genes were the ones identified using the AtGenExpress microarray datasets (Figure S5). ABA, abscisic acid; ACC, ethlyene; BL, brassinolide; GA, gibberellic acid; IAA, indole acetic acid; MJ, methyl jasmonate. (B) Representative key regulatory enzymes and hormonal signaling genes among the top 200 most strongly enriched targets of SEP3. *GH3.3* encodes an IAA-amido synthase that is involved in auxin homeostasis [78]. *GA1* encodes a key enzyme in giberellic acid biosynthesis [79]. *BRI1* encodes a receptor kinase mediating brassinosteroid signal transduction [80]. *AOC1* and *AOC2* gene products catalyze an essential step in jasmonic acid biosynthesis [81]. (C) Different steps in the auxin signaling pathway targeted by SEP3. PID (kinase) and PIN4 (auxin efflux carrier) are important for auxin transport [47,82]. *ARF3* (*ETT*) and *ARF8* are members of the auxin response factor (ARF) family of transcription factors [49,83]. *MIR167A* is one of the genomic loci encoding for a miRNA targeting ARF6 and ARF8 [84]. *IAA4* is an auxin-induced gene [85]; its gene product possibly acts as antagonist of ARF transcription factors. In (B) and (C), the TAIR annotation of the genomic loci is shown at the bottom of each panel. If the genomic locus is shown above the scale, it is in forward orientation, and if it is in the bottom of the scale, it is in reverse orientation. The scale division corresponds to 2,000 nt. wt, wild type.

The characterization of overrepresented GO categories and transcription factor families suggests that SEP3 is involved in the regulation of hormonal signaling. In order to further understand this link, we analyzed the overlap between the potential direct downstream targets of SEP3 and genes regulated by different hormones [42]. We found that genes regulated by auxin, gibberellic acid, and brassinosteroids were most represented among SEP3 targets, but also genes responding to other hormones were enriched (Figure 8A). Among the top 200 genes targeted by SEP3 are several enzymes involved in hormone biosynthesis (e.g., *GA1* and *AOC2*), signaling (e.g., *BRI1*), or homeostasis (e.g., *GH3.3)* (Figure 8B)*. GH3.3* is involved in auxin homeostasis and has been found to be up-regulated as a later response of carpel and stamen induction by AG [26].

Since auxin signaling was consistently found to be overrepresented among our potential direct SEP3 targets, we further analyzed SEP3 binding patterns at auxin-related genes (Figure 8C). Most Aux-IAA genes showed similar SEP3 binding patterns, with a peak close to the transcriptional start site (similar to *GH3.3*). Next, to genes involved in auxin transport (e.g., *PIN4* and *PID*) and auxin response factors with known roles in flower and fruit development (e.g., *ARF3*, *ARF6*, and *ARF8*), a miRNA167 locus, which controls *ARF6* and *ARF8* expression, is also found among the targets.

### Novel Roles of SEP3 Unraveled by Dominant Repression of Direct Target Genes

SEPALLATA genes have shown to be crucial for the specification of floral meristem and organ identities [6,43]. However, the redundancy among MADS box genes makes the functional characterization of members of this gene family difficult [44]. Indeed, our analysis of potential downstream targets of SEP3 suggests so far unknown links to other developmental and hormonal processes in flower development. Since SEP3 acts mostly as a transcriptional activator, we can alternatively study the functions of this protein by replacing endogenous SEP3 function with that of a chimeric repressor version of SEP3. For this purpose, we fused the genomic coding region of SEP3 to the EAR (ERF-associated amphiphilic repression) domain under the control of a basic SEP3 promoter (−960 bp) [45]. This chimeric repressor blocks the activation of direct target genes of the transcription factor complexes in which SEP3 is present.

**Figure 9:**
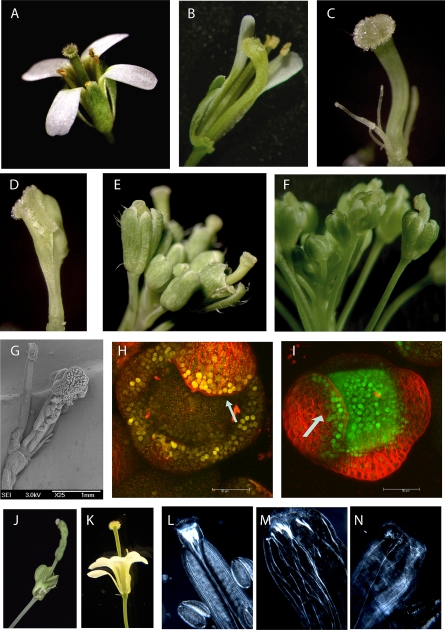
Role of SEP3 in Auxin Signaling (A) Wild-type *Arabidopsis* flower. (B) *ett* mutant flower, two sepals and petals removed. (C and D) Flowers of *SEP3-EAR* plants (*pSEP3::SEP3-EAR* in *sep3–1* mutant) with strong mutant phenotype, sepals (and filamentous organs in [D]) were removed to reveal the inner organs. (E) Inflorescence of *SEP3-EAR* plant. (F) Inflorescence of *ett-1* mutant. (G) SEM picture of a flower from a *SEP3-EAR* plant revealing abnormal ovule placentation and enlarged stigmatic tissue (sepals were removed). (H and I) Localization of nuclear localized DR5::YFP (yellow) in a floral meristem (H). Localization of SEP3-GFP driven from its own promoter in a floral meristem (I). Arrows indicate similar localization of the DR5 marker and SEP3 in sepal tips. White bar indicates 30 μm. (J) Flower of a *sep1 sep2/SEP2 sep3* mutant plant with stalked carpel. (K) *pid* mutant flower with stalked carpel. (L–N) Venation patterns in wild-type (L) and SEP3-EAR carpels (M) and (N).

Our transgenic approach indicated novel functions of SEP3 in addition to supporting previously proposed roles: transgenic lines with a strong phenotype showed delayed flowering (unpublished data), reduced number and size of floral organs, as well as defects in organ differentiation (Figure 9) and identity. While petals were mostly absent in these plants, the stamen number was strongly reduced, and the stamens were often reduced to filamentous carpelloid structures or fused with the carpels (Figures 9C and 9D). Plants with strong phenotypes were male and female sterile. The carpel of severely affected lines showed severe growth defects: the size of the ovary was greatly reduced or it was even missing, while the gynophore at the bottom and the style at the top of the gynoecium were enlarged. Ovule placentation was abaxialized in a variable manner (Figure 9G). The aberrant carpel morphology closely resembles the phenotype of mutants impaired in auxin biosynthesis or signaling [18]. The carpel phenotypes are similar to those of *pin1* [46], *pinoid* (*pid*) [47,48], or *arf3/ett* mutants [49,50,51] (Figure 9B and 9F, versus 9D and 9E). In *sep1 sep2 sep3* triple-mutant plants, elongated gynophores similar to the *pid* mutant can be observed (Figure 9J and 9K). The receptor kinase PID determines the polar localization of auxin efflux (PIN) proteins, whereas ARF3, along with other ARF transcription factors, mediates auxin response at the gene regulatory level.

Our ChIP-SEQ data suggest multiple links of SEP3 and the auxin signaling pathway: ARF genes and their antagonists, the AUX-IAA factors, as well as ARF-controlling microRNA loci are bound by SEP3 as revealed by the ChIP-SEQ experiments. Also, a genomic region downstream of the *PID* locus is targeted by SEP3, both in wild type and in the *ag* mutant. In agreement with a role of SEP3 regulating these genes, expression microarray data of developmental time series suggest that a majority of ARF transcription factor genes bound by SEP3, as well as *PID* and a smaller number of AUX-IAA genes, are up-regulated in reproductive meristems and young floral stages, in a similar fashion to SEP3 itself (Figure S6).

The enrichment of auxin response elements (ARF binding sites) in the SEP3 ChIP-SEQ peaks suggests that SEP3 cooperates with ARF proteins in target gene regulation, thus the downstream targets of these complexes could be the target of repression by the SEP3-EAR protein. The need for cofactors in auxin response is illustrated by the finding that (positive) auxin response, as measured by the DR5 promoter, is only found in a subset of SEP3-expressing cells. Whereas the expression of SEP3 in undifferentiated meristematic tissues is broader than that of the DR5 marker, the expression domains overlap mostly in growing organs such as the tips of growing sepals (Figure 9H and 9I). DR5 expression has been shown to be also dependent on brassinosteroid signaling, which suggests a complex pattern of upstream regulation of auxin response [52].

## Discussion

In order to determine genome-wide binding sites of the MADS-box transcription factor SEP3, we performed ChIP-SEQ and ChIP-CHIP experiments and found a high correlation between the datasets, although the ChIP-SEQ approach provides a higher resolution of the binding pattern. The ChIP data demonstrate that the floral MADS-box transcription factor SEP3 bind to thousands of regions in the *Arabidopsis* genome. Thus, it apparently acts as global regulator of gene expression during the various stages of floral development from floral meristem initiation to maturity. Also, other MADS-box transcription factors bind to thousands of sites in the *Arabidopsis* genome (AP1; K. Kaufmann and G. C. Angenent, unpublished data), indicating that these transcription factors are key regulators controlling the expression of other regulatory genes and structural genes.

### Functional Relevance of Binding Events

Despite the large number of genomic binding sites, only a small fraction of the potential binding sites that are present in the genome, represented by a CArG box, are indeed bound by SEP3. de Folter and Angenent (2006) [12] calculated that the *Arabidopsis* genome contains far more CArG boxes than the roughly 30,000 genes in the genome, whereas our ChIP-SEQ experiments resulted in enriched binding sites in about 3,400 genes (FDR <0.001). This indicates that the binding of SEP3 to DNA sequences is highly selective. Most binding sites are located in promoters or other regions with potentially regulatory functions, such as introns. These findings suggest that the majority of significant protein–DNA interactions identified in ChIP-SEQ experiments are likely to be relevant. The functional importance of the DNA-binding events detected in our ChIP-SEQ experiments is also supported by the finding that there is enrichment for specific GO annotations among the targets of SEP3.

The question remains whether all of the enriched DNA regions bound by SEP3 are relevant for gene regulation. Since the plant material used for our ChIP-SEQ experiments comprises different developmental stages, a direct correlation of DNA-binding events and stage-specific changes in gene expression is difficult. However, our comparison of the ChIP-SEQ data to comprehensive gene expression microarray data suggest that the majority of the genes that are bound by SEP3 *in planta* are also differentially expressed during flower development in a temporal and/or spatial fashion.

In contrast to the strongly bound regions, the functional relevance of weakly enriched regions detected in ChIP-SEQ is much less clear. They might represent transient interactions of the transcription factors with DNA [53], or DNA binding in only a few cells, resulting in a high dilution with tissues that lack the interaction. In a comparable study to decipher binding sites of transcription factors during *Drosophila* embryo development, Li et al. came to the conclusion that a significant proportion of the poorly bound regions are most likely nonfunctional [15]. These regions corresponded to genes that were poorly modulated in expression, and peaks were often located outside regulatory sequences. It is possible that the weakly enriched regions in our SEP3 ChIP experiment also contain a high proportion of inactive binding sites.

### Determinants of In Vivo DNA Binding

Although in vitro studies have revealed consensus binding sites for many transcription factors, including MADS-domain proteins [12,32], it remains unknown what DNA sequence or chromosomal context determines DNA binding site recognition by MADS-box transcription factors in vivo. Our results indicate that the sequence of the cognate binding site, the presence of multiple binding sites, and binding sites for non-MADS cofactors play roles in binding site recognition. The SEP3 ChIP data revealed that not all DNA sequences corresponding to the canonical consensus motifs CC[A/T]_6_GG and CC[A/T]_7_G/C[A/T]_7_GG can serve as functional binding sites for SEP3 and associated MADS-domain proteins *in planta*. Instead, we found significant enrichment for particular types of half-sites in the SEP3 ChIP-SEQ data. In addition, there is clear interdependence between the sequence of individual half-sites, and between half-sites and surrounding bases. Thus, commonly used consensus sequences and position weight matrices, which assume independency of nucleotide positions of a binding site, are highly oversimplified. The enrichment for certain half-sites may reflect the recognition site for SEP3, while the other half of the binding site could match the recognition site of the dimer partner of SEP3. It is known that SEP3 is able to dimerize with many other MADS-domain proteins in yeast assays [8] and *in planta* (K. Kaufmann and G. C. Angenent, unpublished data), which makes it difficult to determine *the* consensus CArG box sequence for SEP3. Combining ChIP-SEQ data obtained from different MADS dimer partners (e.g., SEP3 and AP1) and determining the overlap in binding sites will elucidate the recognition sites for particular MADS-box dimers. We found that there is a clear overlap of target genes in wild type compared to the *ag* mutant, although there are differences in number of binding sites and binding affinity at individual sites. Although at this point we cannot exclude that the wild-type binding sites are enriched for perianth-specific targets, these results support the hypothesis that different MADS-domain protein complexes (e.g., AG-SEP3 and SEP3-AP1) may bind to overlapping sets of target genes, but regulate them in a different way, for instance by recruiting different sets of cofactors leading to differences in activation or repression of genes. In line with the idea that cofactors play a role in differential regulation of downstream targets, it has been shown that SEP3 and AP1 can recruit the corepressor SEUSS, and that this complex acts to repress AG expression in petal development [11]. Our finding that individual peaks are more likely to be affected by loss of AG than target genes makes it also possible that different higher-order complexes may bind to subsets of binding sites present in the promoter. Considering that plant MADS-domain proteins differ in their DNA bending characteristics as determined by gel retardation experiments [28], different protein complexes may have specific effects on the structural properties of the promoter and by that influence target gene expression.

The interplay between different proteins implicates that the dynamics in the floral developmental network is strongly dependent on relative quantities and affinities of individual homeotic proteins competing for common protein interaction partners and key downstream targets. The large number of targets suggests that floral homeotic protein complexes globally control and modify the genetic programs that are active in all plant organs, so that only a limited number of targets would be expected to be unique for the individual floral homeotic protein complexes.

In addition to CArG box elements, we found several other transcription factors consensus DNA-binding sequences to be enriched in the center of the peaks, suggesting that these transcription factors act in a combinatorial fashion with MADS-domain proteins in common regulatory modules. Until now, there has been very limited information about interactions between plant MADS-domain proteins and other transcription factors, and this rare information is based on artificial yeast and/or in vitro protein interaction data. The types of transcription factors whose binding sites are enriched in the peaks link MADS-domain proteins with other cellular, developmental, and hormonal pathways. TCP transcription factors have been shown to be important for cell growth [54–56], and ARF transcription factors are key mediators of auxin response (reviewed in [57]). Whether there are direct protein interactions between these classes of transcription factors still needs to be resolved.

In addition to primary sequence characteristics, the accessibility of binding sites, and thus the chromatin structure, can influence the recruitment of MADS-box transcription factors to specific sites in the genome. We observed a strong enrichment of binding sites close to the transcriptional start site of genes, suggesting that the recognition sites are not randomly distributed in the genome. Assuming that any small sequence motif is randomly distributed in the genome, it suggests that not only the primary sequence itself, but also the position of the *cis*-element is relevant for transcription factor binding. Chromatin remodeling, which is active throughout plant development, is likely affecting the accessibility of transcription factors and transcriptional activity. It occurs during postembryonic developmental transitions (e.g., flowering) and is required for maintenance of meristematic cell identity as well as for the formation of organ primordia, with different chromatin factors acting at different stages ([58] and references therein). Current models of gene regulation suggest that chromatin remodeling and transcription factor binding dynamically alternate due to transient exposure of DNA by displacement of nucleosomes ([59] and references therein). MADS-box transcription factors form large complexes *in planta* (K. Kaufmann and G. C. Angenent, unpublished data), making it even possible that there is a direct interaction between these transcription factors and chromatin remodeling factors. Interesting in this respect is that we identified four out of five members of ATP-dependent chromatin remodeling complexes as targets of SEP3 in the ChIP experiments, suggesting an interdependence relationship.

### Auxin Signaling as a Target Pathway of SEP3

Our ChIP-SEQ data indicate that there are multiple direct molecular links between floral homeotic genes and hormonal pathways. Interestingly, ARF genes are targets of SEP3 but could also be coregulators, suggesting that autoregulatory circuits exist involving members of unrelated transcription factor families.

In addition to specifying floral meristem and organ identity, an important role of SEP and AP1-like proteins is to trigger organ growth. The *ap1 cal* double mutant produces multiple undifferentiated meristems, in which organ outgrowth and differentiation are impaired [60]. Similar mutant phenotypes arise by combining *ap1* and *sep* mutant alleles, suggesting partial redundancy between members of these subfamilies of MADS-box genes [43]. This hypothesis of a mutual role of AP1/CAL and SEP proteins in organ outgrowth provides a link to auxin-mediated organ development. Interestingly, the expression of SEP3 fused to the EAR suppression domain led to phenotypes that mimicked developmental aberrations observed in *pin1*, *pid*, or *ettin* (*arf3*) mutants or plants treated with the auxin transport inhibitor NPA [61]. These plants are characterized by defects in lateral organ outgrowth (floral buds and floral organs) and a pistil lacking a functional ovary. Although we observed variable homeotic conversions in SEP3-EAR expressing plants, which would be expected from the down-regulation of SEP3 function, the majority of plants was only affected in outgrowth and differentiation of the floral organs. The mode of action of the SEP3-EAR fusion protein and how it interferes with outgrowth without affecting the homeotic function remain to be studied further. A possible explanation could be that auxin homeostasis is more sensitive to the dominant repression by the SEP3-EAR protein than the floral homeotic functions. Alternatively, SEP3-EAR may suppress ARF function by interacting with ARF proteins in a larger transcriptional complex, a model that is supported by our SEP3-ChIP experiments.

### Transcriptional Networks in Flower Development

Developmental transcription networks are composed of positive and negative regulatory loops, which often unite into larger transcription units. Positive feedback loops that are made up of two transcription factors regulating each other result in a robust expression in response to a transient developmental signal in order to establish and maintain a developmental program [62]. However, negative regulation is also very important in developmental processes to rapidly switch from one program to another (e.g., phase transitions).

**Figure 10:**
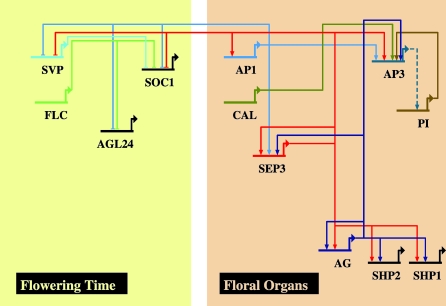
Autoregulatory Network of MADS Box Transcription Factors in *Arabidopsis* Flower Development The network was visualized using the BioTapestry program [55]. According to our results, SEP3 is involved in the direct repression of flowering time genes, as well as in the activation of floral homeotic genes by binding to their respective promoters. The regulation of *PI* by AP3 is not confirmed by experimental approaches so far and might be indirect (dashed line).

The expression of MADS-box genes is often regulated by transcription complexes composed of their own gene products. One of the earliest examples for this phenomenon was the finding that floral homeotic genes responsible for the formation of petals and stamens (the B class genes in the ABC model) are up-regulated by dimers consisting of the encoded proteins [63]. More examples of autoregulatory feedback loops in the MADS-box gene family were reported afterwards. Also, *SEP* genes and their protein products are part of these regulatory networks [6]. The ability of the SEP3 protein to form many different dimer combinations [8] and the interaction of SEP3 protein and other floral homeotic proteins in larger protein complexes [10,64] illustrate that SEP3 is a key component in the network. It acts as a hub by linking various developmental programs that occur in the inflorescence and floral meristems and at later stages during organ differentiation. Our ChIP-SEQ data demonstrate that SEP3 is indeed able to bind to the promoters of many floral homeotic genes, supporting the conclusion that SEP3 is a key regulator of flower development (Figure 10).

From Figure 10, it is apparent that most MADS-box genes are targeted by a combination of several other MADS-domain proteins. The finding that most of these MADS-domain proteins also physically interact with each other suggests that combinatorial interactions of floral homeotic MADS-domain proteins and SEP3 protein are required for multiple positive autoregulatory feedback and feedforward loops. These mutual interdependencies may have evolved in order to enable stable organ-specific expression patterns by attenuating stochastic fluctuations in expression levels that may be more frequent if regulation was mediated by single proteins instead of heteromeric protein complexes.

Previously, negative feedback loops were suggested between AP1, which is a protein interaction partner of SEP3, and some of the flowering-time MADS-box genes to prevent the expression of these vegetative factors in the flower [65]. Our data also suggest that SEP3 has a role in repression of genes that control flowering time (e.g., *SOC1* and *AGL24*). In line with this idea, binding sites of SEP3 and AP1 overlap at the *SOC1* promoter ([65] and our ChIP-SEQ data). Interestingly, the binding sites also partially overlap with those of positive regulators at the *SOC1* locus ([66] and our ChIP-SEQ data). It is possible that positively and negatively acting factors compete for the same binding sites. Since SEP3 is also able to interact with SOC1, it is also possible that a SEP3-SOC1 protein dimer is important for negative autoregulation of the SOC1 locus in floral meristems [8]. In general, direct negative (auto)regulatory feedback loops may enable an efficient and persistent switch between developmental phases, i.e., from inflorescence identity to floral identity.

Transcription factor complexes and their target genes are major components of transcriptional cascades that drive plant developmental processes. Once we have genome-wide datasets describing more of these interactions, we can integrate the data into transcriptional networks and models describing the interactions of the components of the network and predicting the outcome of modulation of the biological system [67,68]. Here, we have shown that ChIP-SEQ is a powerful tool providing these essential datasets to address questions in plant biology. Our findings suggest that many direct regulatory interactions exist in plant developmental networks. Additional information is needed, in particular the dynamics of the interactions in time and space. Furthermore, not only DNA–protein interactions, but also protein–protein interactions of transcription factors need to be resolved for a better understanding of developmental processes in complex organisms.

## Materials and Methods

### Plant material and growing conditions.

Arabidopsis thaliana, wild-type (Col-0), *sep3*-1 (Col-0), and *ag*-1 (Ler) mutant plants were grown under standard greenhouse conditions (20 °C, long-day light regime: 16-h light, 8-h dark cycle). Flower material was harvested from primary and secondary inflorescences of 5–7-wk-old plants.

### Chromatin immunoprecipitation (ChIP).

ChIP experiments were performed essentially as described in [69]. For the IP, we used an antibody raised against a C-terminal peptide of SEP3. The antibody was tested in western analyses on plant extracts of wild-type and *sep3* mutant plants. Total extracts were produced using a standard protocol [70]; nuclei extracts were produced following the protocol that was used for the ChIP experiments, only that the flower material was not fixed. Cross-reaction with other SEPALLATA MADS-domain proteins was tested in western blots using proteins produced by in vitro translation.

### Sample preparation for ChIP-SEQ and ChIP-CHIP, tiling array hybridization, Solexa sequencing.

Linker annealing, amplification, and gel purification for the Solexa sequencing were essentially performed as instructed by the Illumina protocol with small modifications. The gel purification was done after the amplification step. We used individual, complete ChIP samples for each amplification reaction for the wild-type and *ag-*1 mutant samples. For the *sep3*-1 mutant sample (negative control), we pooled the DNA of three different ChIP experiments to obtain sufficient material for amplification. The amplified material was subjected to Solexa sequencing following Illumina's instructions. The sequence datasets were submitted to Gene Expression Omnibus (GEO) (accession number GSE14600).

For the amplification of the ChIP-DNA for ChIP-CHIP, we used a protocol published by [71] with modifications. The amplified DNA was partially digested with DNAse I (fragments <150 bp) and labeled with biotin. For the control experiment, we used unamplified, sheared chromatin from the same biological sample as the ChIP-DNA (“input DNA”). The DNA was fragmented and labeled in the same way as the ChIP-DNA. The labeled samples were hybridized to GeneChip *Arabidopsis* Tiling 1.0R Arrays (Affymetrix) as biological duplicates. Tiling array data were submitted to GEO (accession number GSE14635).

### Processing of the tiling array data.

Enrichments in ChIP sample hybridizations relative to input were calculated from raw intensity (CEL) files using a nonparametric statistical method implemented in the Affymetrix Tiling Analysis Software (TAS) [72]. Biological replicates were combined in the analysis. Once the significance (*p*-value) was obtained, we define as ChIP-CHIP peaks the genomic regions with a *p*-value lower than 0.05, and not separated by more than 100 bp with the highest *p*-value not higher than 0.05.

### Primary statistical analysis of the ChIP-SEQ data.

The 35- or 36-nucleotide (nt) reads were mapped to the unmasked *Arabidopsis* reference genome (ATH1.1con.01222004; ftp://ftp.arabidopsis.org/) using the SOAP software [73], allowing a maximum of two mismatches and no gaps. Iteratively, one base was discarded from the end of the nonmapped reads until the reads were uniquely mapped or fell below a minimum read length of 30 nt. Only uniquely mapped reads were retained. In order to recover the average length of the original DNA fragments that were subjected to Solexa sequencing, the reads were extended directionally to 300 nt.

The data analysis was aimed at a comparison of the enrichment found in the wild-type plant and, independently, in the *ag-1* mutant, against the enrichment found in the *sep3-*1 mutant treated as a negative control. For this, data concerning positions of the mapped reads were transformed into numbers characterizing all nucleotide positions in the genome in the following way. Define *x_is_*, where *i* is the nucleotide position and *s* = 1,2 for the examined and control sample respectively, as the minimum of the counts of extended mapped reads that overlap at the position *i* on the forward and on the reverse strand in the sample *s*. This value is a conservative estimate of the representation of the nucleotide *i* in the sequenced samples, supported by both the strands independently. By the transformation

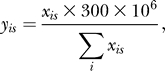
observations *y_is_* independent of the number of sequenced reads were obtained. The examined sample values *y_i_*_1_ were then normalized with respect to the mean and variance of the distribution of control values *y_i_*_2_. For the comparison of the observed examined and control counts at position *i*, the one-sided test based on the Poisson distribution was made according to the probability (test statistic) formula

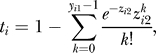
where *z_i_*_2_ is the maximum of *y_i_*_2_ and the global coverage obtained for the control whose value is the product of the number and the length of the extended mapped reads divided by the mappable genome length. All genomic regions consisting of nucleotides characterized by calculated probability values smaller than 0.05 and not interrupted by a gap of 100 nt or more were identified and assumed to contain a candidate enrichment peak. For presentation purposes, the calculated probabilities were transformed into scores of −log_e_(*t_i_*), and the maximum score value for each candidate peak was used to test the significance.

Permutation tests were used to estimate the FDR for the peaks. For this, each mapped read was considered as having the label “sample” when it belonged to the examined sample and “control” if it belonged to the control sample. To obtain the distribution of the test statistic under the null hypothesis of no differences between the examined and the control samples, the labels of the reads were randomly permuted, and for each permutation, the methodology explained above to test differences in distribution was applied. The permutations were run until at least 65,000 test statistic values for calculation of the null distribution were obtained.

### Positional characterization of ChIP-SEQ peaks.

All peaks were characterized by their location with respect to the annotated genes (as described in the TAIR7_GFF3_genes.gff file, ftp://ftp.arabidopsis.org/). The gene affected by binding at each peak was selected by the following algorithm. First, for all peaks, the affected gene was selected as the one with the peak inside it and the minimum distance to the start. For the peaks outside of the genes, the affected gene was then selected as the one with the peak in its 3,000-bp upstream or 1,000-bp downstream region and the minimum distance to the start or end, respectively. Thus, it was assumed that a DNA-binding event affects the closest neighboring gene, which results in a conservative estimate of the number of genes controlled by each of the transcription factors.

The genes affected by binding inside them were then characterized with respect to the precise annotation of the position of the affecting peak, by considering three categories: 5′ UTR, 3′ UTR, other exon regions (equivalent to CDS for protein coding genes), or “not annotated.” This characterization was done on the basis of the first available splicing variant for a gene; in practice, this meant using variant 1 for most of the genes and variant 2 for some.

### De novo motif discovery.

Default parameters for MatrixREDUCE [30] and MEME [36] algorithms were used in order to identify motifs de novo. Sequences 500 bp upstream and downstream of the maximum score position for significant peaks (FDR <0.001) were obtained from the *Arabidopsis* reference genome (ATH1.1con.01222005; ftp://ftp.arabidopsis.org/). Repeat and low-complexity regions were eliminated using RepeatMasker (A. F. A. Smit, R. Hubley, and P. Green, RepeatMasker at http://repeatmasker.org). Sequence affinity logo representations were prepared using AffinityLogo [30]. The MatrixReduce algorithm uses genome-wide occupancy/affinity data for a transcription factor and associated nucleotide sequences to discover the sequence-specific binding affinity of the transcription factor. It utilizes a statistical-mechanical model to describe the relationship between the nucleotide sequences and the occupancy/affinity-related score, therefore avoiding the need of selecting any background sequence model. In contrast, MEME only uses the occupancy/affinity-related score to define a group of nucleotide sequences from which a general binding site consensus will be obtained.

### Search for known transcription factor binding sites.

Perfect match motif consensus sequences were located in the 1,000-bp region around the maximum score position (defined as “peak area”) for significant peaks (FDR <0.001) using a perl script and the *Arabidopsis* reference genome. The motif consensus sequences were obtained from AGRIS and Transfac databases [37,74,75]. Each consensus sequence was associated with the score value of the corresponding ChIP-SEQ peak in order to calculate enrichment.

### DNA binding site characterization in genomic regions bound by SEP3.

For each significant peak, the nucleotide sequence 500 bp around the position of the maximum peak score location were extracted and associated with the peak score value. To obtain the proportion of peaks with a given DNA binding site consensus at a given peak score threshold level, among the nucleotide sequences associated with a peak score value bigger than the threshold level, the proportion of sequences with at least one DNA binding site consensus was calculated for the sample and control set. Two control sets were generated: (1) one control set was generated randomly permutating the nucleotide sequences and the peak score value, and therefore destroying any relationship between them, and (2) another control set was generated permutating the nucleotides within their sequence for each nucleotide sequence independently. Each set was characterized by the number and location (regarding the center of the sequence) of the DNA consensus binding sites studied. We considered binding sites to be overrepresented in the ChIP-SEQ data only when they were enriched relative to both controls. For simplicity, only control 2 is shown in Figures 1 and 3.

The proportion of the distance of the DNA binding site consensus to the peak score location was calculated as the distance from the center position of the DNA consensus to the peak score location for each peak with a score bigger than the corresponding threshold at a given FDR level. Nonoverlapping distance ranges of 50 bp were considered to calculate the proportion of distance values within each range among the total number of distance values considered. All graphs were generated with R software.

### Identification of regulatory modules.

ExPlain [35] was used to identify transcription factor binding sites in the sequence probes generated by the ChIP-SEQ experiment, and to generate promoter models of regulation modules composed of more than one binding site. All CArG box position weight matrices from Transfac [37] were collected into a set, and thresholds were set to minimize false negatives. The algorithm MATCH [37] was used to identify putative sites matching the CArG box matrices in the profile. Site frequencies were compared to a control sequence set consisting of randomly sampled *Arabidopsis* promoters, assuming a binomial distribution. The algorithm Composite Module Analyst (CMA, [76]) was used to identify transcription modules overrepresented in the query dataset, based on the binding site prediction by MATCH. CMA parameters were set to find modules composed of one or more pairs of sites separated by 10, 11,..., 200 nt. CMA evaluates a fitness score, which includes functions measuring normality, *t*-test, site orientation and distance, sequence match score, and model complexity.

### Analysis of the distance distribution between CArG box site pairs.

In order to establish whether CArG box pairs presented conserved distances between them, a comparison between the distance distribution between sites found in the real data and those found in two control datasets was performed. The control sets generated consisted of (1) real promoter sequences from genes chosen at random from the *Arabidopsis* genome, and (2) random permutations of the sequences described above. The control or background set using real promoters (1) makes a more stringent test, because real binding sites, including CArG boxes, can still be found in the sequence. This test shows the specific selection of a given distance between CArG box site pairs in the SEP3-dependent gene's promoters, when compared against other real promoter sequences. The second background set, made out of randomly permuted sequences (2), presents sites generated by the random variation of the genome composition, without (biological) selection, so that this comparison shows the selection of distances between these sites, when compared with an absolutely random site and distance distribution. The MATCH algorithm was applied to these three sequence sets, using the CArG box profile described above, and the positions of the matching sites collected. The distance between adjacent sites was calculated and the distributions were compared using a binomial test, to identify overrepresented distances.

### Gene ontology analysis.

Analysis for GO term enrichment was carried out using the AMIGO server [77] for the top 1,000 genes of the ChIP-SEQ dataset (Database version 2008-05-15). Only GO terms with more than 20 annotated loci were taken into account. We considered the most specific categories, ones that were found to be enriched by AMIGO, for further analyses. To study in more detail the enrichment for the top-five most significant, most specific GO terms (GO:0008610 “lipid biosynthetic process,” GO:0045449 “regulation of transcription,” GO:0009908 “flower development,” GO:0048513 “organ development,” and GO:0009733 “response to auxin stimulus”) and the nonenriched term GO:0051179 “localization,” each gene was associated with the maximum score value among the peaks that were affecting it. We mapped each gene to a GO term if the gene belongs to one of these GO terms or its children, using the Arabidopsis Information Resource (TAIR) ATH-GO-GOSLIM 2008-05-10.

### Gene expression microarray analysis.

We downloaded log_2_ gcRMA normalized expression values for experiments of interest from the AtGenExpress developmental atlas [39]. Control probe sets and probe sets matching no or several loci in the *Arabidopsis* genome were ignored in the analysis. After back-transforming the log_2_ expression values to original scale, a two-sided Student test statistic was applied to test differential expression between sample and control. These genes with a *p*-value lower than 0.01 were considered as differentially expressed.

### Generation of the SEP3-EAR construct and transgenic plants.

To generate the dominant repressor, we used the SRDX domain [45], which is a modified version of the EAR domain of the SUPERMAN protein. The promoter and coding region including introns of SEP3 was amplified from genomic. We added the SRDX domain in two subsequent round of PCR reactions. The primer sequences are available on request. The PCR product recombined into pDONR207, and subsequently into the destination vector pFP101-35SGa. The destination vector was obtained digesting the plasmid pFP101 with BamHI and HindIII, filled in with Klenow enzyme and blunt-end ligated with Gateway cassetteB. The construct was transformed into the *sep3*-1 mutant plants and Col-O wild-type plants. SEP3-EAR expression in wild type and *sep3* mutant showed similar phenotypes; however. we focused our phenotypic analysis on *pSEP3:SEP3-EAR* in the *sep3* mutant.

### SEP3-GR induction and quantitative gene expression analysis.

Transgenic seedlings were germinated and grown on plates without DEX for 10 d and transferred to medium containing 10 mM DEX for 8 h or 1 d, respectively. Alternatively, they were directly germinated on DEX medium and grown for 10 d. cDNA synthesis was produced using the iScript cDNA synthesis kit, and qPCRs were performed using the SYBR green I–based system from BioRad following the manufacturer's instructions. Two technical and two independent biological replicates were analyzed using the MyIQ program. The data were normalized with two reference genes (*TUB* and *EF*). All primers are available as supplementary information (Table S6).

### Coexpression analysis.

The expression values for development stages (floral transition to flowers stage 12; AtGenExpress experiments 6, 8, 29, 31, 32, 33, and 39) were obtained as explained in “Gene expression microarray analysis” above. After back-transforming the log_2_ expression values to original scale, the Spearman rank correlation test was applied to check the coexpression for each gene on the array with SEP3. For each peak score threshold, the proportion of genes that correlate with SEP3 expression at different control levels (0.05, 0.01, 0.005, and 0.001) among the genes affected by at least one ChIP-SEQ peak was calculated. In a similar way, the proportion of genes that correlate with SEP3 expression among all the genes was calculated.

## Supporting Information

Figure S1 Western Blot Analysis (A) Western blot of in vitro–translated MADS-domain proteins probed with the SEP3 antibody used for the ChIP experiments (upper panel). The same proteins were detected using Streptavidin-Alkaline Phosphatase to test for expression of the biotin-labeled proteins (loading control; lower panel).
(B) Western blot on plant extracts. Left: comparison of SEP3 antibody signal in total and nuclei extracts. Right: detection of SEP3 in wild-type and sep3 mutant lines. Riken line PST1193 has an insertion in the upstream region of SEP3 near the transcriptional start. Riken line PST20678 has an insertion in an exon.
(1.57 MB PDF)

Figure S2 Comparison ChIP-SEQ and ChIP-CHIP Data (A) Histogram of distances between the maximum score positions of significant peaks in ChIP-SEQ and ChIP-CHIP: 90.7% of the peaks in ChIP-CHIP have a ChIP-SEQ peak nearby (distance <1,000 bp).
(B) Distance between the top 4,000 ChIP-SEQ peaks and the top 4,000 ChIP-CHIP peaks as a function of the sum of their ranks. Peaks with low rank sums (high significance in ChIP-SEQ and ChIP-CHIP) are mostly a short distance from each other. Peaks with the higher rank sums are also mostly overlapping or only a short distance apart; however, more variation is observed.
(C) Box-plot of the widths of ChIP-SEQ peaks for different ranks (groups of 1,000). The lower the rank, the more significant is a peak.
(D) Box-plot of the widths of ChIP-CHIP peaks for different ranks. In (C) and (D), only the lowest 10,000 ranks are shown.
(E) Scatter plot of the average number of overlapping extended reads at each nucleotide position based on the raw sequence data for the two sequencing replicates of the SEP3 wild-type ChIP sample. The average was obtained over nonoverlapping windows of length 5,000 bp (Pearson correlation, 0.97).
(F) Scatter plot of rank positions of ChIP-SEQ and ChIP-CHIP peaks. For each ChIP-SEQ peak, the closest ChIP-CHIP peak was identified (at a distance not greater than 500 bp). The scatter plot shows the relationship between the peak rank positions in ChIP-CHIP and ChIP-SEQ. Only the 14,000 most significant peaks are shown. The peaks that are in a similar position in CHIP-SEQ and in ChIP-CHIP (not farther apart than 500 bp) have a similar rank position in both experiments. This relationship is stronger for the more significant peaks. In order to minimize the effects of technical differences related to the different experimental platforms, we did not consider the value of the test statistics for each peak, but rather its rank (the most significant peak has a rank of one).
(G) Scatter plot of the average number of overlapping extended reads at each nucleotide position based on the raw sequence data for the two biological replicates of the SEP3 wild-type ChIP sample. The average was obtained over nonoverlapping windows of length 5,000 bp (Pearson correlation, 0.81).
(H) Distance between the top 4,000 ChIP-SEQ peaks of two biological replicates as a function of the sum of their ranks. Peaks with low rank sums (high significance in ChIP-SEQ and ChIP-CHIP) are mostly a short distance from each other. Peaks with higher rank sums are also mostly overlapping or a short distance apart.
(914 KB PDF)

Figure S3 Peak Position within Genomic Features as a Function of SEP3 ChIP-SEQ Peak Score (A) Enrichment of peaks within promoters (black line, up to 3 kb upstream) and downstream regions (green, up to 1 kb downstream) with increasing ChIP-SEQ score. Peaks within genes (red line) and peaks without any neighboring gene (blue line) are also shown in the graph.
(B) Preferred position of peaks within introns (blue line), 5′ UTRs (black line) and 3′ UTRs (green line). Peaks in coding sequences (red line) are also indicated in the graph.
(C) Position of the ChIP-SEQ peak maxima relative to the transcriptional start of genes (zero position). Enrichment of peaks (as indicated by proportion) depends on the FDR used.
(D) As in (C) but now for the end of genes (zero position is end of the gene).
(539 KB PDF)

Figure S4 Enrichment of MYC and C2H2 (ID1) Binding Sites with Increasing Peak Score Threshold The frequency of ID1 binding sites in the genome and in the ChIP-SEQ data is very low, the enrichment however is supported by two types of controls. Black line: ChIP-SEQ data (wild-type); red line: Permutation of score and sequence; blue line: Randomization of sequences.
(537 KB PDF)

Figure S5 Expression Analysis of Potential Direct SEP3 Targets Compared to Genome-Wide Data (A) Gene expression analysis of different floral homeotic mutants (meristematic tissue up to floral stage 7 for *lfy-*12, *ap1-*15, *ap2-*6, *ap3-*6, and *ag-*12) or different developmental stages (meristem before floral transition up to flowers of stage 12).
(B) Enrichment of genes correlated in expression with SEP3. The Spearman rank correlation *p*-value was calculated as described in Material and Methods.
(582 KB PDF)

Figure S6 Developmental Time Series Expression Data of Auxin-Related Genes Targeted by SEP3 Expression in vegetative above-ground organs and in flowers and reproductive meristems is shown. The expression of *PIN1*, which is itself not a target of SEP3, is also visualized.
(607 KB PDF)

Table S1 Overview of Results from Solexa Sequencing and Mapping of the Reads (A) Results of Solexa sequencing and mapping of the reads to the genome.
(B) Reads mapped to chromosomes (in percent) in relation to chromosome length.
(46 KB DOC)

Table S2 List of Genes Targeted by SEP3 in Wild Type and *agamous* Mutant Based on the ChIP-SEQ Experiments at FDR <0.001 The file also contains a table with data on differential expression of potential target genes in the *ag* mutant compared to wild type.
(921 KB XLS)

Table S3 Genomic Positions of ChIP-SEQ Peaks in Different Categories of Genes Genomic positions of ChIP-SEQ peaks relative to closest neighboring genomic loci in wild type (A) and *ag* mutant (B), and within genes in wild type (C) and *ag* mutant (D).
(80 KB DOC)

Table S4 Frequency of Half-Sites and Complete Sequences Corresponding to the Consensus CC[A/T]_6_GG and C[A/T]_7_GG Overrepresentation was calculated given the observed number of these sequences in wild-type and *agamous* ChIP-SEQ peaks compared to their genome-wide frequencies using a binomial test.
(41 KB XLS)

Table S5 Dependencies between Nucleotide Positions in CArG Boxes of Type CC[A/T]_6_GG The *p*-values of the chi-square test were used to measure dependence. The table shows the dependence in −log_e_ scale. Only values bigger than −log_e_(0.05) are shown.
(17 KB XLS)

Table S6 List of Primers Used in the Real-Time RT-PCR Experiments (61 KB XLS)

## Abbreviations

ChIP: chromatin immunoprecipitation
ChIP-CHIP: chromatin immunoprecipitation coupled with DNA microarray hybridization
ChIP-SEQ: chromatin immunoprecipitation coupled with Solexa sequencing
FDR: false discovery rate
GO: gene ontology
