# Supplementary material for: Target Genes of the MADS Transcription Factor SEPALLATA3: Integration of Developmental and Hormonal Pathways in the Arabidopsis Flower
Source: PLoS Biol. 2009 Apr 21;7(4):e1000090. doi: 10.1371/journal.pbio.1000090 (PMC2671559; doi:10.1371/journal.pbio.1000090)
Supplement: Supplementary file 2 [file pbio.1000090.sg002.pdf]

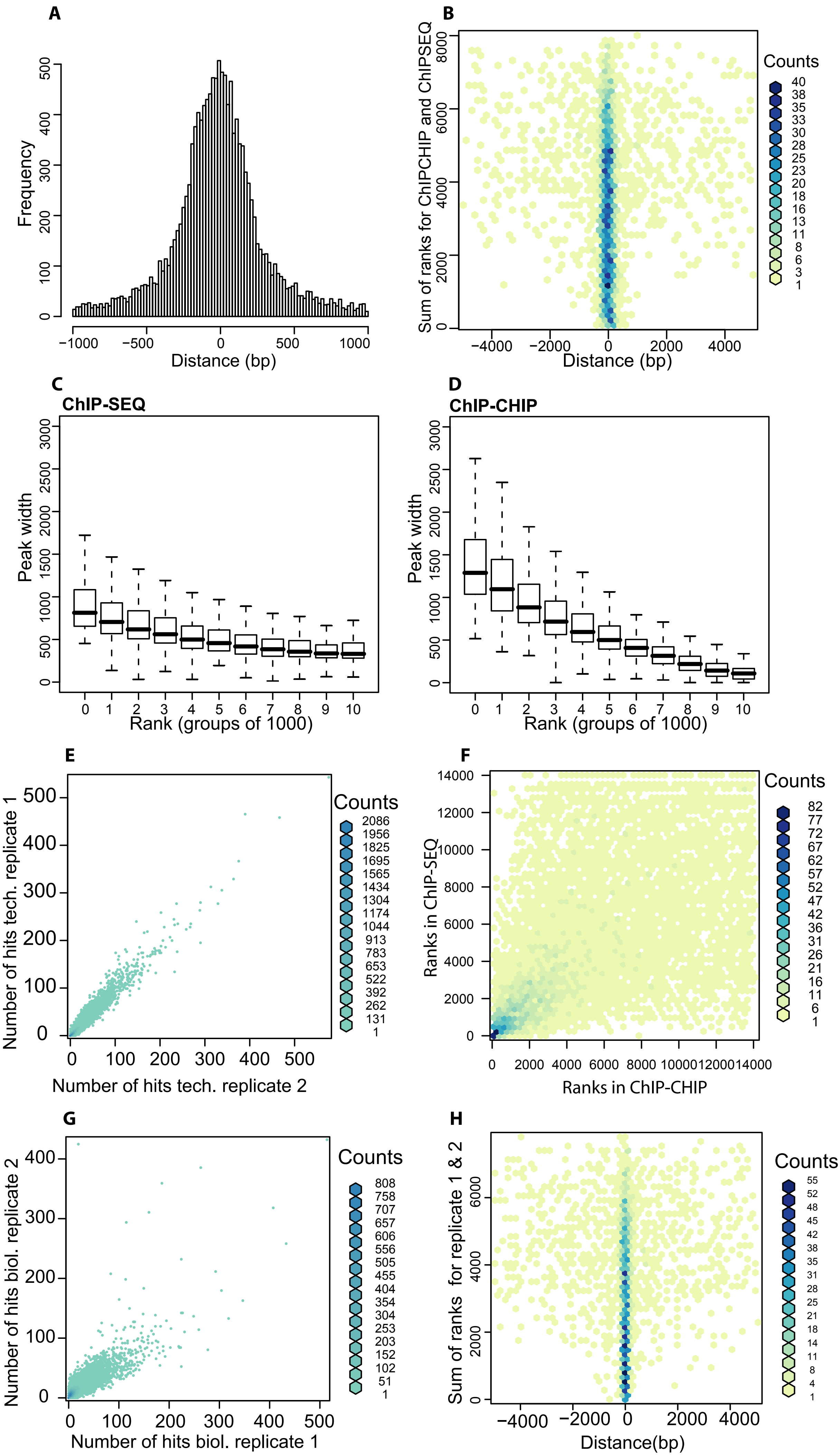
A
Frequency
0
100
200
300
400
500
−1000
−500
0
500
1000
Distance (bp)
B
Sum of ranks for ChIPCHIP and ChIPSEQ
0
2000
4000
6000
8000
−4000
−2000
0
2000
4000
Distance (bp)
Counts
40
38
35
33
30
28
25
23
20
18
16
13
11
8
6
3
1
C
ChIP-SEQ
Peak width
0
500
1000
1500
2000
2500
3000
0
1
2
3
4
5
6
7
8
9
10
Rank (groups of 1000)
D
ChIP-CHIP
Peak width
0
500
1000
1500
2000
2500
3000
0
1
2
3
4
5
6
7
8
9
10
Rank (groups of 1000)
E
Number of hits tech. replicate 1
0
100
200
300
400
500
0
100
200
300
400
500
Number of hits tech. replicate 2
Counts
2086
1956
1825
1695
1565
1434
1304
1174
1044
913
783
653
522
392
262
131
1
F
Ranks in ChIP-SEQ
0
2000
4000
6000
8000
10000
12000
14000
0
2000
4000
6000
8000
10000
12000
14000
Ranks in ChIP-CHIP
Counts
82
77
72
67
62
57
52
47
42
36
31
26
21
16
11
6
1
G
Number of hits biol. replicate 2
0
100
200
300
400
0
100
200
300
400
500
Number of hits biol. replicate 1
Counts
808
758
707
657
606
556
505
455
404
354
304
253
203
152
102
51
1
H
Sum of ranks for replicate 1 & 2
0
2000
4000
6000
−4000
−2000
0
2000
4000
Distance(bp)
Counts
55
52
48
45
42
38
35
31
28
25
21
18
14
11
8
4
1
