# Supplementary material for: Target Genes of the MADS Transcription Factor SEPALLATA3: Integration of Developmental and Hormonal Pathways in the Arabidopsis Flower
Source: PLoS Biol. 2009 Apr 21;7(4):e1000090. doi: 10.1371/journal.pbio.1000090 (PMC2671559; doi:10.1371/journal.pbio.1000090)
Supplement: Supplementary file 3 [file pbio.1000090.sg003.pdf]

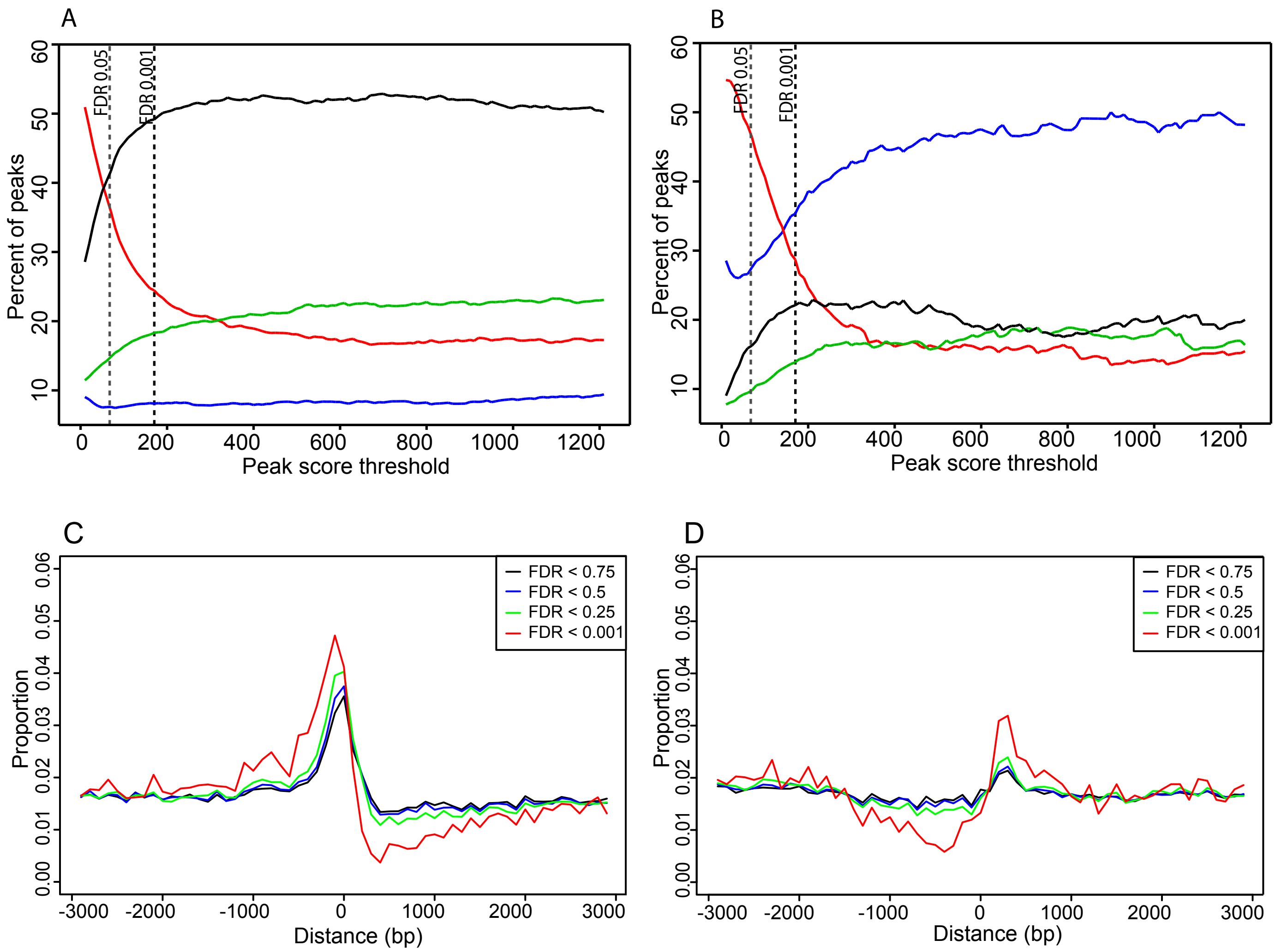

A
B
C
D
FDR 0.05
FDR 0.001
Percent of peaks
Peak score threshold
Proportion
Distance (bp)
FDR < 0.75
FDR < 0.5
FDR < 0.25
FDR < 0.001
