# Supplementary material for: Target Genes of the MADS Transcription Factor SEPALLATA3: Integration of Developmental and Hormonal Pathways in the Arabidopsis Flower
Source: PLoS Biol. 2009 Apr 21;7(4):e1000090. doi: 10.1371/journal.pbio.1000090 (PMC2671559; doi:10.1371/journal.pbio.1000090)
Supplement: Supplementary file 4 [file pbio.1000090.sg004.pdf]

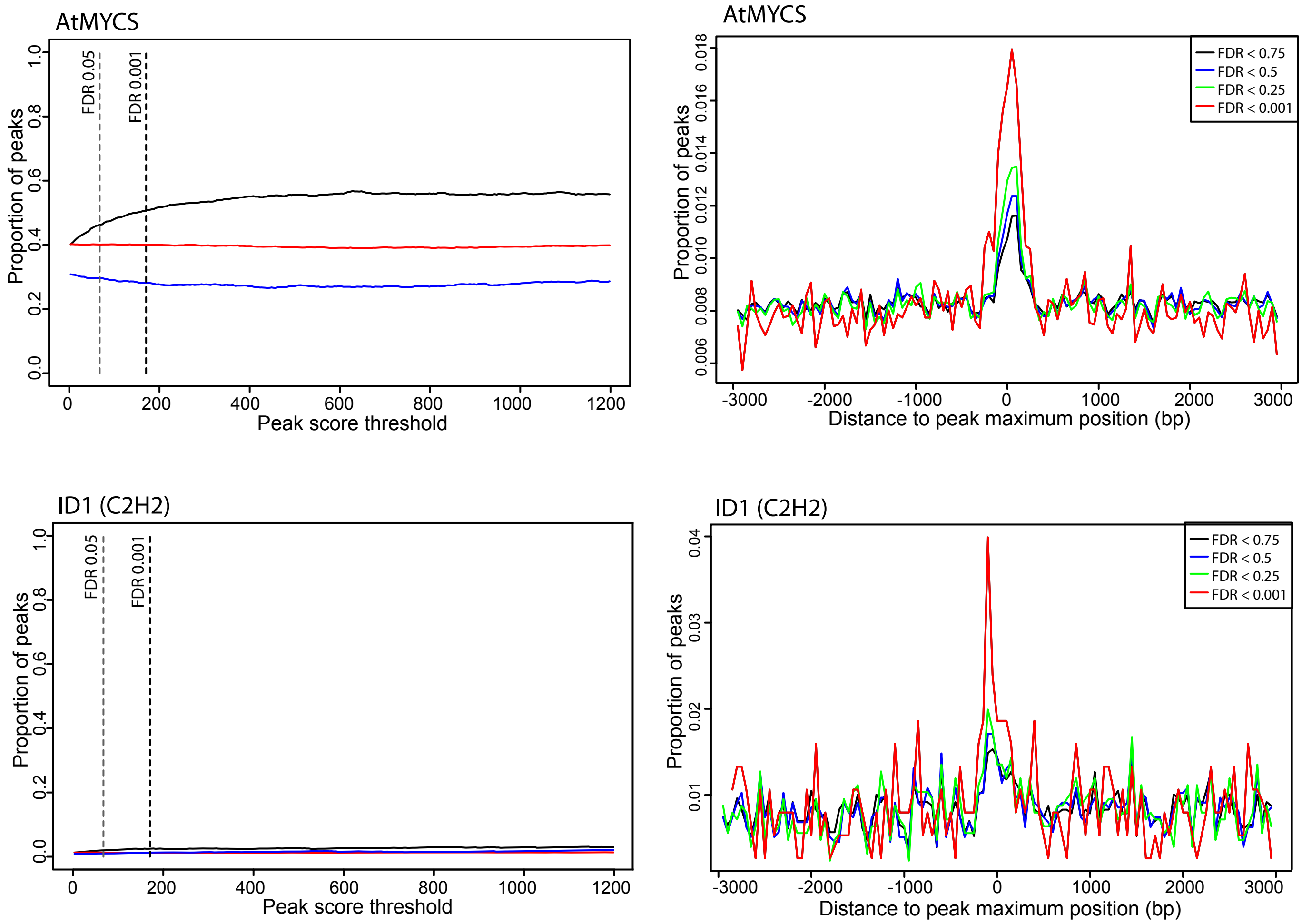
AtMYCS
Proportion of peaks
1.0
0.8
0.6
0.4
0.2
0.0
FDR 0.05
FDR 0.001
0
200
400
600
800
1000
1200
Peak score threshold
AtMYCS
Proportion of peaks
0.018
0.016
0.014
0.012
0.010
0.008
0.006
FDR < 0.75
FDR < 0.5
FDR < 0.25
FDR < 0.001
-3000
-2000
-1000
0
1000
2000
3000
Distance to peak maximum position (bp)
ID1 (C2H2)
Proportion of peaks
1.0
0.8
0.6
0.4
0.2
0.0
FDR 0.05
FDR 0.001
0
200
400
600
800
1000
1200
Peak score threshold
ID1 (C2H2)
Proportion of peaks
0.04
0.03
0.02
0.01
FDR < 0.75
FDR < 0.5
FDR < 0.25
FDR < 0.001
-3000
-2000
-1000
0
1000
2000
3000
Distance to peak maximum position (bp)
