# Supplementary material for: Target Genes of the MADS Transcription Factor SEPALLATA3: Integration of Developmental and Hormonal Pathways in the Arabidopsis Flower
Source: PLoS Biol. 2009 Apr 21;7(4):e1000090. doi: 10.1371/journal.pbio.1000090 (PMC2671559; doi:10.1371/journal.pbio.1000090)

A. Global gene expression analysis

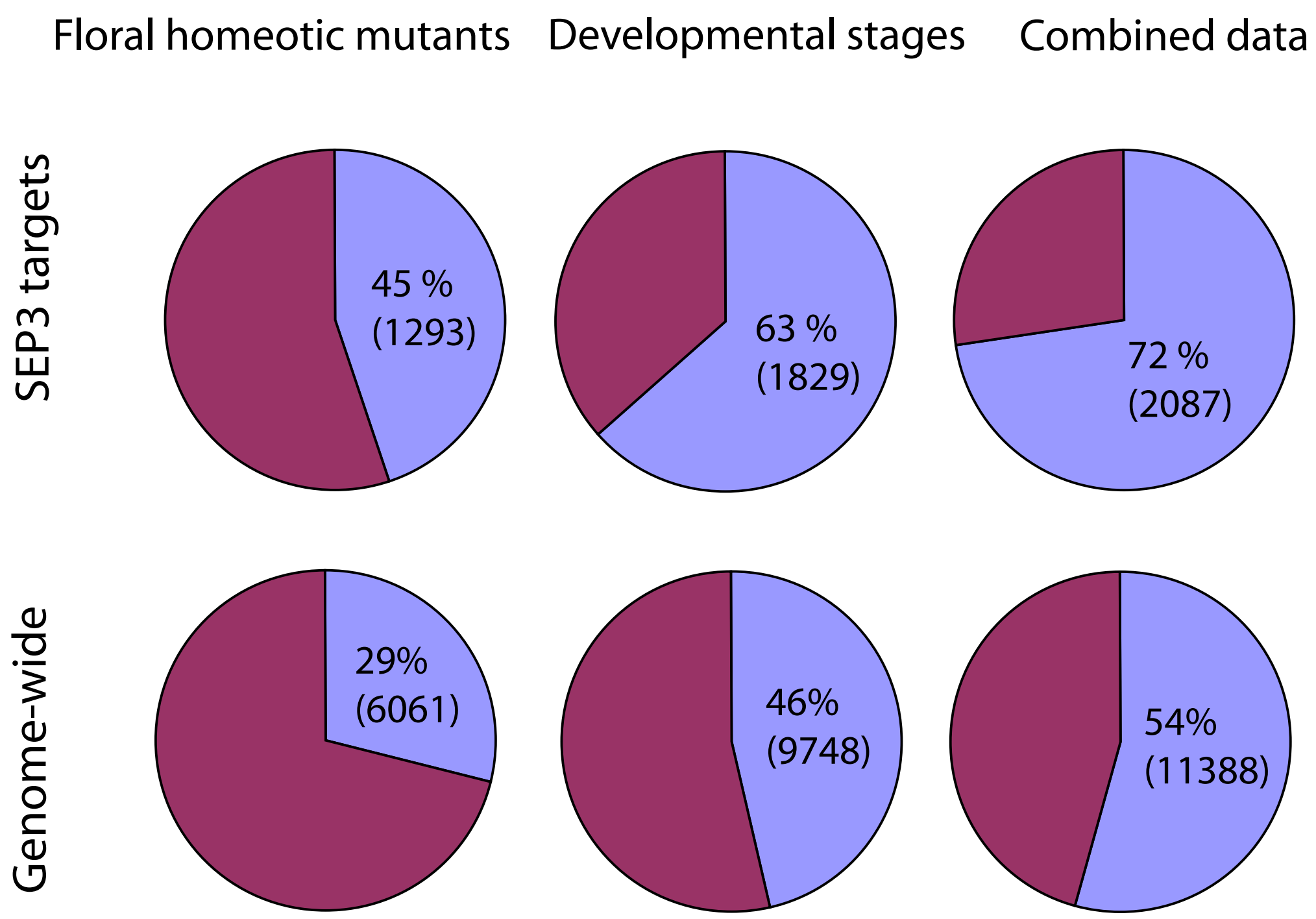

B. Genes correlated with correlated expression to SEP3

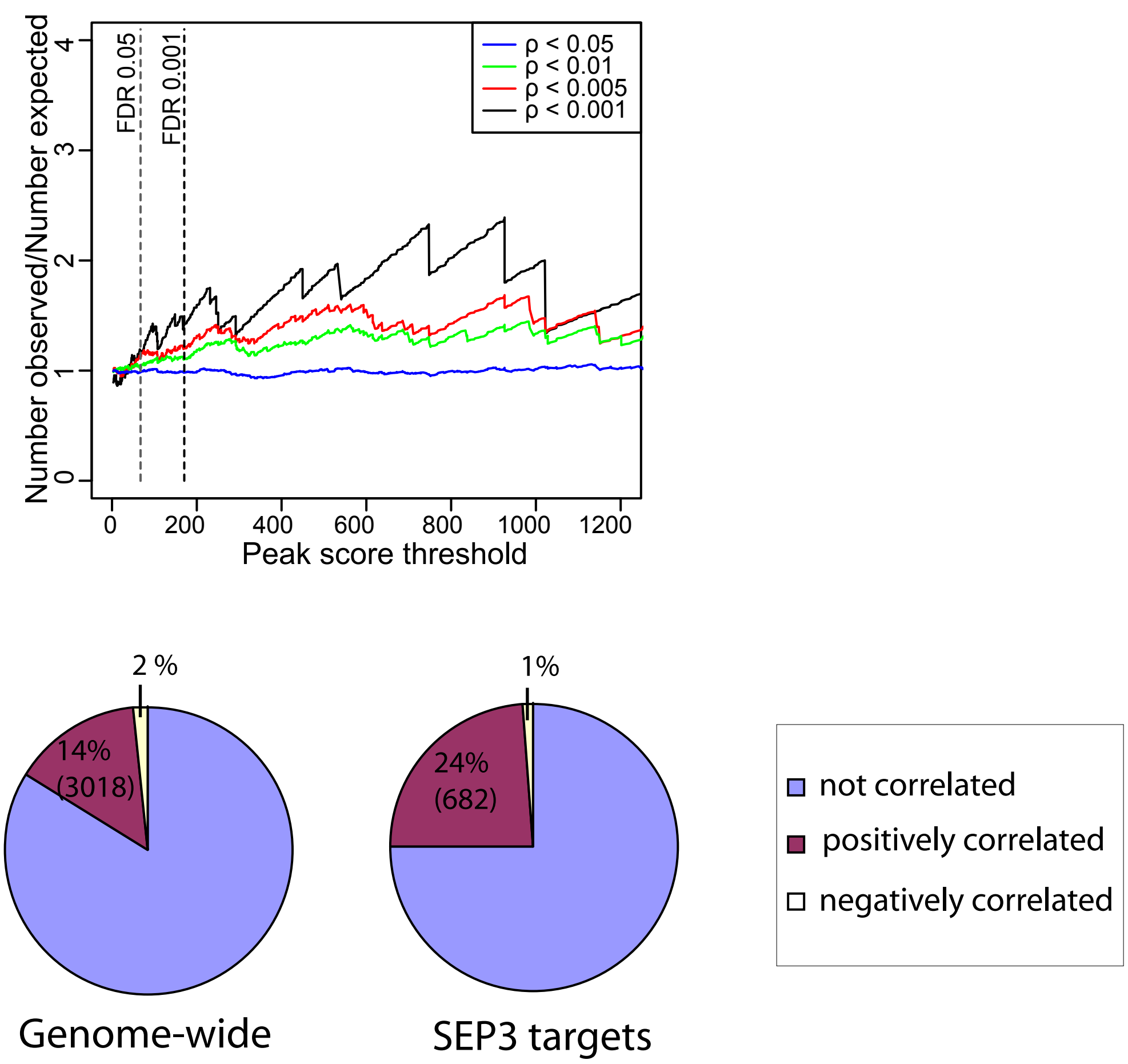

Supplement: Supplementary file 5 [file pbio.1000090.sg005.pdf]
