# Supplementary material for: Target Genes of the MADS Transcription Factor SEPALLATA3: Integration of Developmental and Hormonal Pathways in the Arabidopsis Flower
Source: PLoS Biol. 2009 Apr 21;7(4):e1000090. doi: 10.1371/journal.pbio.1000090 (PMC2671559; doi:10.1371/journal.pbio.1000090)
Supplement: Supplementary file 6 [file pbio.1000090.sg006.pdf]

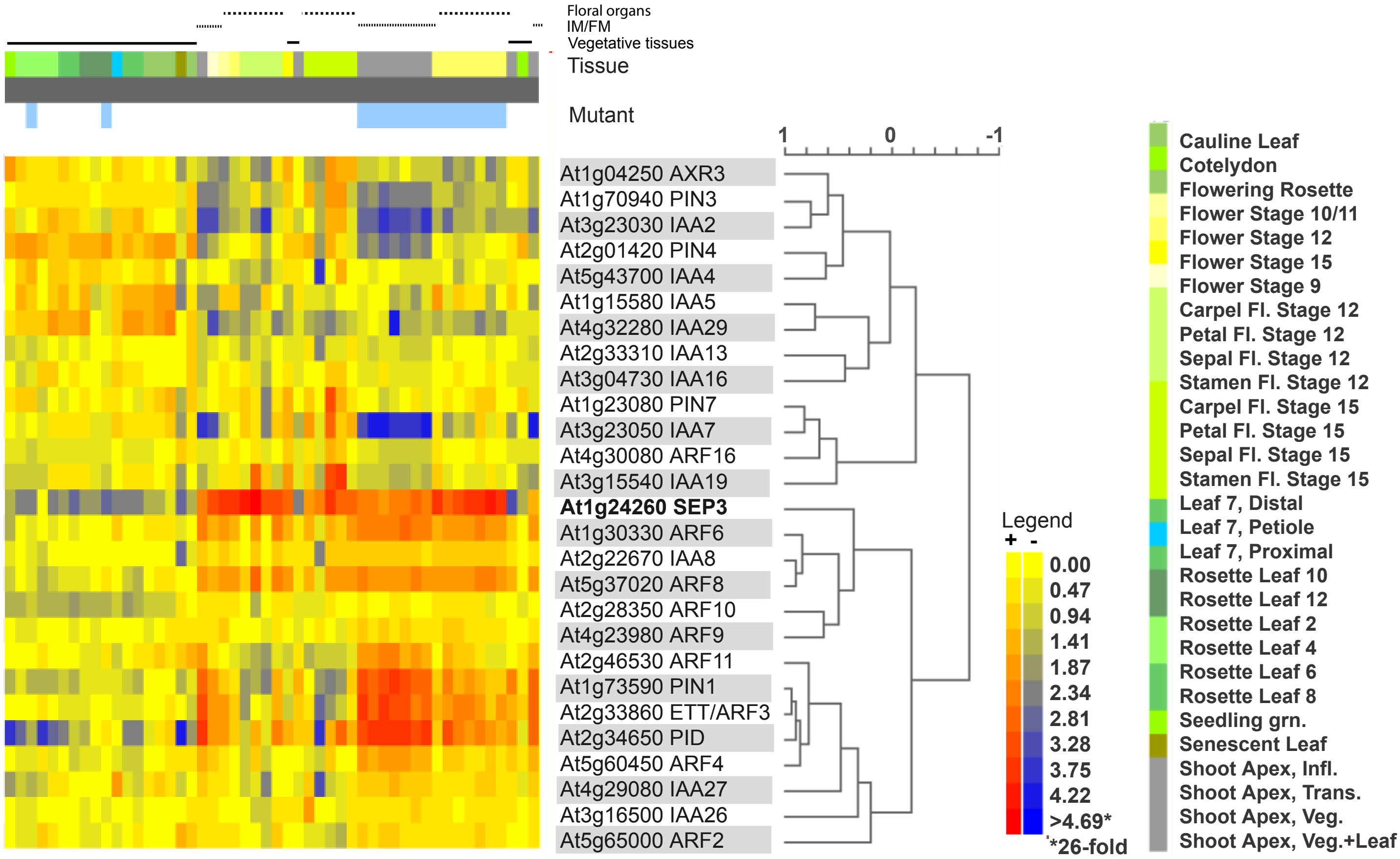

Floral organs
IM/FM
Vegetative tissues
Tissue
Mutant
1
0
-1
At1g04250 AXR3
At1g70940 PIN3
At3g23030 IAA2
At2g01420 PIN4
At5g43700 IAA4
At1g15580 IAA5
At4g32280 IAA29
At2g33310 IAA13
At3g04730 IAA16
At1g23080 PIN7
At3g23050 IAA7
At4g30080 ARF16
At3g15540 IAA19
At1g24260 SEP3
At1g30330 ARF6
At2g22670 IAA8
At5g37020 ARF8
At2g28350 ARF10
At4g23980 ARF9
At2g46530 ARF11
At1g73590 PIN1
At2g33860 ETT/ARF3
At2g34650 PID
At5g60450 ARF4
At4g29080 IAA27
At3g16500 IAA26
At5g65000 ARF2
Legend
+
-
0.00
0.47
0.94
1.41
1.87
2.34
2.81
3.28
3.75
4.22
>4.69*
*26-fold
Cauline Leaf
Cotelydon
Flowering Rosette
Flower Stage 10/11
Flower Stage 12
Flower Stage 15
Flower Stage 9
Carpel Fl. Stage 12
Petal Fl. Stage 12
Sepal Fl. Stage 12
Stamen Fl. Stage 12
Carpel Fl. Stage 15
Petal Fl. Stage 15
Sepal Fl. Stage 15
Stamen Fl. Stage 15
Leaf 7, Distal
Leaf 7, Petiole
Leaf 7, Proximal
Rosette Leaf 10
Rosette Leaf 12
Rosette Leaf 2
Rosette Leaf 4
Rosette Leaf 6
Rosette Leaf 8
Seedling grn.
Senescent Leaf
Shoot Apex, Infl.
Shoot Apex, Trans.
Shoot Apex, Veg.
Shoot Apex, Veg.+Leaf
