# Supplementary material for: Target Genes of the MADS Transcription Factor SEPALLATA3: Integration of Developmental and Hormonal Pathways in the Arabidopsis Flower
Source: PLoS Biol. 2009 Apr 21;7(4):e1000090. doi: 10.1371/journal.pbio.1000090 (PMC2671559; doi:10.1371/journal.pbio.1000090)
Supplement: Supplementary file 7 [file pbio.1000090.st001.doc]

**Supplementary Table S1A**. Summary of sequencing and mapping

|  | wild-type 1 | wild-type 2 | *agamous* | *sep3*-1 |
| --- | --- | --- | --- | --- |
| Total number of reads | 7 455 490 | 3 404 244* | 4 247 537 | 4 067 720 |
| Number of mapped reads | 2 387 714 | 736 082 | 1 332 260 | 1 812 533 |
| as % of total reads | 32.03 | 21.62 | 31.37 | 44.56 |
| Number of mapped reads to chromosomes 1-5 | 2 315 719 | 681 883 | 1 271 190 | 1 640 977 |
| as % of total reads | 31.06 | 20.03 | 29.93 | 40.34 |
| Number of reads mapped to chromosomes |  |  |  |  |
| 1 | 599 480 | 174 279 | 328 281 | 403 922 |
| 2 | 367 336 | 111 202 | 201 826 | 271 778 |
| 3 | 456 913 | 134 002 | 252 922 | 340 341 |
| 4 | 361 502 | 106 101 | 199 530 | 253 469 |
| 5 | 530 488 | 156 299 | 288 631 | 371 467 |
| Chloroplast | 57 609 | 47 884 | 49 028 | 137 979 |
| Mitochondrial | 14 386 | 6 315 | 12 042 | 33 577 |
| Number of reads mapped as |  |  |  |  |
| forward | 1 190 149 (49.84%) | 341 139 (49.99%) | 665 535 (49.96) | 906 164 (49.99%) |
| reverse | 1 197 565 (51.16%) | 340 744 (50.01%) | 666 725 (50.04) | 906 369 (50.01%) |

* prefiltered for adapter-only sequences

**Supplementary Table S1B.** Reads mapped to chromosomes (in %) in relation to chromosome length

| Number of reads mapped to chromosomes | wil-type | *Wkk11* | *sep3*-1 | Chromosome length  (% of genome) |
| --- | --- | --- | --- | --- |
| 1 | 25.11 | 23.68 | 22.28 | 25.42 |
| 2 | 15.38 | 15.11 | 14.99 | 16.46 |
| 3 | 19.14 | 18.20 | 18.78 | 19.61 |
| 4 | 15.14 | 14.41 | 13.98 | 15.53 |
| 5 | 22.22 | 21.23 | 20.49 | 22.55 |
| Chloroplast | 2.41 | 6.51 | 7.61 | 0.13 |
| Mitochondrial | 0.60 | 0.86 | 1.85 | 0.31 |
