# Supplementary material for: Target Genes of the MADS Transcription Factor SEPALLATA3: Integration of Developmental and Hormonal Pathways in the Arabidopsis Flower
Source: PLoS Biol. 2009 Apr 21;7(4):e1000090. doi: 10.1371/journal.pbio.1000090 (PMC2671559; doi:10.1371/journal.pbio.1000090)
Supplement: Supplementary file 8 [file pbio.1000090.st003.doc]

Table S3. Numbers of genes affected by peaks, in different categories of genes depending on positions within or near these genes, for FDR < 0.001.

A. wild-type

| **Gene category** | **No. of genes affected in the region** | | | **Total number of affected genes** | **As %** | **% genes in genome** |
| --- | --- | --- | --- | --- | --- | --- |
|  | upstream | inside | downstream |  |  |  |
| miRNA | 18 | 1 | 6 | 23 | 0.66 | 0.40 |
| other_RNA | 12 | 11 | 9 | 27 | 0.78 | 0.79 |
| protein_coding_gene | 1963 | 963 | 756 | 3393 | 97.64 | 96.22 |
| rRNA | 0 | 1 | 1 | 1 | 0.03 | 0.01 |
| snoRNA | 1 | 0 | 0 | 1 | 0.03 | 0.25 |
| snRNA | 0 | 0 | 0 | 0 | 0 | 0.05 |
| tRNA | 20 | 0 | 10 | 30 | 0.86 | 2.26 |
| total | 2014 | 976 | 782 | 3475 | 100 | 100.00 |

B. *ag* mutant

| **Gene category** | **No. of genes affected in the region** | | | **Total number of affected genes** | **As %** | **% genes in genome** |
| --- | --- | --- | --- | --- | --- | --- |
|  | upstream | inside | downstream |  |  |  |
| miRNA | 10 | 1 | 3 | 14 | 0.58 | 0.40 |
| other_RNA | 8 | 9 | 5 | 20 | 0.83 | 0.79 |
| protein_coding_gene | 1297 | 735 | 474 | 2370 | 97.77 | 96.22 |
| rRNA | 2 | 0 | 1 | 2 | 0.08 | 0.01 |
| snoRNA | 1 | 0 | 0 | 1 | 0.04 | 0.25 |
| snRNA | 0 | 0 | 0 | 0 | 0 | 0.05 |
| tRNA | 13 | 0 | 4 | 17 | 0.70 | 2.26 |
| total | 1331 | 745 | 487 | 2424 | 100 | 100.00 |

C. wild-type within genes

| **Gene category** | **No. of genes affected inside in the region annotated as** | | | | **Total number of affected genes** |
| --- | --- | --- | --- | --- | --- |
|  | 5’UTR | exon | 3’UTR | not annotated |  |
| miRNA |  |  |  | 1 | 1 |
| other_RNA |  | 5 |  | 6 | 11 |
| protein_coding_gene | 229 | 263 | 146 | 351 | 963 |
| rRNA |  | 1 |  |  | 1 |
| snoRNA |  |  |  |  |  |
| snRNA |  |  |  |  |  |
| tRNA |  |  |  |  |  |
| total | 229 | 269 | 146 | 358 | 976 |

D. *ag* mutant within genes

| **Gene category** | **No. of genes affected inside in the region annotated as** | | | | **Total number of affected genes** |
| --- | --- | --- | --- | --- | --- |
|  | 5’UTR | exon | 3’UTR | not annotated |  |
| miRNA |  | 1 |  |  | 1 |
| other_RNA |  | 8 |  | 1 | 9 |
| protein_coding_gene | 221 | 220 | 77 | 231 | 735 |
| rRNA |  |  |  |  |  |
| snoRNA |  |  |  |  |  |
| snRNA |  |  |  |  |  |
| tRNA |  |  |  |  |  |
| total | 221 | 229 | 77 | 232 | 745 |
