# Supplementary material for: Target Genes of the MADS Transcription Factor SEPALLATA3: Integration of Developmental and Hormonal Pathways in the Arabidopsis Flower
Source: PLoS Biol. 2009 Apr 21;7(4):e1000090. doi: 10.1371/journal.pbio.1000090 (PMC2671559; doi:10.1371/journal.pbio.1000090)
Supplement: Supplementary file 9 [file pbio.1000090.st006.doc]

| **Gene** | **Primer name** | **Sequence** |
| --- | --- | --- |
| AP1 | 2219 | AAATCCAGCATCCTTACATGCTCTC |
|  | 2220 | CAGTTCGAGATCATTCCTCCTCATT |
| SVP | 2221 | ACTAACGGAAGAGAACGAGCGACTT |
|  | 2222 | GGCGTTAGTAATAGACTCCGACGAC |
| SHP1 | 2223 | TGATACAAGGGACGACAGTTTACGA |
|  | 2224 | AGAATTGCTGATTCGGTTCAAGAAG |
| SHP2 | 2225 | AAATCGAGCTGCAAAACGATAACAT |
|  | 2226 | GTAACACCCGACTCGTAAACTGTCC |
| AGL24 | 2227 | CGGAATTGGTGGATGAGAATAAGAG |
|  | 2228 | GTTCCACTGTCGTAGCTTGACACAT |
| FUL | 2229 | AAGGACAATTAGTCCAATGCTCCAA |
|  | 2230 | CAACTCTCTCCACAAAGCCATCTCT |
| AGL16 | 2231 | ACATGAAAAGGTTTCAGAGGTCGAG |
|  | 2232 | AGATGGACATGTTCGTTCGAGGTAT |
| AGL15 | 2233 | GTCAAGCGATTCAGTGACAACAAAC |
|  | 2234 | CAGAGAACCTTTGTCTTTTGGCTTC |
| AP3 | 2169 | CAGTTCTTGGATACCAAATCGAAGG |
|  | 2170 | AAGGTAATGATGTCAGAGGCAGAGG |
| SOC1 | 2142 | TGAAAGCGAAGTTTGGTCAAATAAGA |
|  | 2143 | TCTTGAAGAACAAGGTAACCCAATGA |
| SEP4 | 1748 | CCAAGTAGATGCATCACTAAGACAA |
|  | 1749 | CTTTCTCCTAAGATCTCTATTGGTTTCCA |
| SEP2 | 1751 | GGACTATACCAATCTCTTGAATGTG |
|  | 1752 | CTTGCACCGTCACAGCCATTTGCT |
| SEP1 | 1753 | CTATACCAGCCTCTTGAATGCA |
|  | 1754 | TGAGCTTGGGTTGTCGCAGTT |
| PI | 1279 | TGTCGAGCACGCCATTGAACAT |
|  | 1280 | CTGGAAAGTGAGTTGCCGTTG |
| SEP3 | 1897 | AAAGAGAGGGATTGATTAAGTGAGAAAGA |
|  | 1899 | GTATCAGGGGCAACAAGATGGAAT |
| AG | 1277 | CTACGAGCAGCTTATGCCACCA |
|  | 1278 | GAGTAATGGTGATTGTTAGGTTGC |
| CAL | 2250 | GATCGCTCATCAGACTTCTCCTTTC |
|  | 2251 | GCCAAGGTAATTGTAAATGGGTTCA |
| STK | 2248 | CCATCATCAAATGGTTAGTGGTTCA |
|  | 2249 | ATTACCAGATCCAGAACCAGCAGTC |
| TUB | 1362 | ACCACTCCTAGCTTTGGTGATCTG |
|  | 1363 | AGGTTCACTGCGAGCTTCCTCA |
| EF | 1364 | TGAGCACGCTCTTCTTGCTTTCA |
|  | 1365 | GGTGGTGGCATCCATCTTGTTACA |
